# Selective Deletion of Heparan Sulfotransferase Enzyme, Ndst1, in Donor Endothelial and Myeloid Precursor Cells Significantly Decreases Acute Allograft Rejection

**DOI:** 10.1038/s41598-018-31779-7

**Published:** 2018-09-07

**Authors:** Hao Chen, Sriram Ambadapadi, Dara Wakefield, Meeyong Bartee, Jordan R. Yaron, Liqiang Zhang, Stephanie A. Archer-Hartmann, Parastoo Azadi, Michelle Burgin, Chad Borges, Donghang Zheng, Kevin Ergle, Vishnu Muppala, Sufi Morshed, Kenneth Rand, William Clapp, Amanda Proudfoot, Alexandra Lucas

**Affiliations:** 1 The Department of Tumor Surgery, Second Hospital of Lanzhou University, Lanzhou, China; 2 Divisions of Cardiovascular Medicine and Rheumatology, Department of Medicine, University of Florida, Gainesville, FL USA; 3 Department of Molecular Genetics and Microbiology, College of Medicine, University of Florida, Gainesville, FL USA; 4 Center for Personalized Diagnostics, and the Center of Immunotherapy, Vaccines and Virotherapy, The Biodesign Institute, Arizona State University, Tempe, AZ USA; 5 Department of Pathology, University of Florida, Gainesville, FL USA; 6 Complex Carbohydrate Research Center, University of Georgia, Athens, GA USA; 7 Novimmune, Geneva, Switzerland

## Abstract

Early damage to transplanted organs initiates excess inflammation that can cause ongoing injury, a leading cause for late graft loss. The endothelial glycocalyx modulates immune reactions and chemokine-mediated haptotaxis, potentially driving graft loss. In prior work, conditional deficiency of the glycocalyx-modifying enzyme N-deacetylase-N-sulfotransferase-1 (Ndst1^f/f^ TekCre^+^) reduced aortic allograft inflammation. Here we investigated modification of heparan sulfate (HS) and chemokine interactions in whole-organ renal allografts. Conditional donor allograft Ndst1 deficiency (*Ndst1*^−/−^; C57Bl/6 background) was compared to systemic treatment with M-T7, a broad-spectrum chemokine-glycosaminoglycan (GAG) inhibitor. Early rejection was significantly reduced in *Ndst1*^−/−^ kidneys engrafted into wildtype BALB/c mice (*Ndst1*^+/+^) and comparable to M-T7 treatment in C57Bl/6 allografts (P < 0.0081). M-T7 lost activity in *Ndst1*^−/−^ allografts^,^ while M-T7 point mutants with modified GAG-chemokine binding displayed a range of anti-rejection activity. CD3+ T cells (P < 0.0001), HS (P < 0.005) and CXC chemokine staining (P < 0.012), gene expression in NFκB and JAK/STAT pathways, and HS and CS disaccharide content were significantly altered with reduced rejection. Transplant of donor allografts with conditional *Ndst1* deficiency exhibit significantly reduced acute rejection, comparable to systemic chemokine-GAG inhibition. Modified disaccharides in engrafted organs correlate with reduced rejection. Altered disaccharides in engrafted organs provide markers for rejection with potential to guide new therapeutic approaches in allograft rejection.

## Introduction

Acute and chronic transplant rejection, with scarring and organ failure, prolong illness, increase mortality and increase the necessity for repeat transplant^1–7^. Organs available for transplant are limited and there is a large, unmet need for new treatments that reduce transplant vasculopathy and rejection. Early rejection is the leading cause of graft loss in the first year post-transplant, while chronic rejection with allograft vascular disease is a leading cause of late graft loss after the first year post-transplantation and some of this ongoing chronic damage is believed to be initiated early after graft implant. Transplant allograft vasculopathy (TAV) causes graft scarring and late loss associated with chronic rejection. Development of chronic rejection with TAV is induced, in part, by both recurrent episodes of acute antibody-mediated immune rejection and also persistent excess inflammation^1–4^. Thus, both changes related to cellular rejection and antibody-mediated rejection have the potential to induce early damage to the graft with long-lasting effects. Some of these inflammatory, non-antibody mediated immune responses are produced by surgical and ischemic injury and infection at the time of transplant, occurring early after engraftment with long lasting effects on organ function^1–10^.

Most treatments for preventing rejection target the immune response of the recipient host and few have investigated directly treating the donor organ prior to transplantation as a method to reduce early damage and ongoing excess inflammation. Local inflammation may be driven by changes in the endothelial layer glycocalyx after injury. Thus, we have postulated that treatments designed to modify the donor tissue glycocalyx content may beneficially alter early innate and acquired immune responses.

Glycosaminoglycans (GAGs) are complex, linear, negatively-charged polymers consisting of repeating subunits of polysaccharide sugars. Heparan sulfate (HS) is the predominant GAG present on the surface of cells, representing a major component of the extracellular matrix, with multiple roles in physiological and pathophysiological processes. GAGs are critical in vascular physiology where they form the glycocalyx, a meshwork of carbohydrates that coats vascular endothelial cells and regulates vascular permeability, acts as a transducer of fluid shear forces, modulates receptor activity and cellular adhesion and/or activation, and provides the substrate for directional chemokine gradient formation to mediate leukocyte chemotaxis and invasion^1,11–15^. GAGs and the endothelial glycocalyx may have a role in tissue graft survival. For example, treatment with a mutant of the CXCL8 (IL-8) chemokine, which has enhanced GAG and reduced chemokine receptor binding, has been reported to reduce early rejection in a rodent transplant model, further supporting a central role for chemokine-GAG interactions in rejection^15–17^. In prior work, reductions in HS binding and HS glycoproteins, such as perlecan, modified acute monocyte chemoattractant protein-1 (MCP-1)-mediated monocyte infiltration in renal ischemia reperfusion injury. Blockade of chemokine-GAG interactions using MC2, a peptide derived from the HS-GAG binding domain of IFN gamma (IFNγ) also reduces inflammation and prolongs dermal graft survival in a mouse model^15–17^. The endothelial glycocalyx therefore has fundamental roles in cellular responses in early rejection, whether cellular or antibody mediated.

Chemokines have proven dual interactions with GAGs and with 7 transmembrane G protein coupled chemokine receptors on immune cells^15–19^. This requisite GAG and receptor interaction presents one mechanism through which modified GAG composition may alter acute transplant injury and rejection. Chemokines activate cells via surface receptors, however, certain chemokines also unexpectedly signal cell activation through cooperative receptor activation via direct GAG interaction, bypassing receptors^7^.

While chemokines have been studied extensively in transplants, the role of GAG interactions is less well defined^16–19^. As noted, the main tissue GAG is heparan sulfate (HS), but the endothelial glycocalyx also contains other GAGs such as chondroitin sulfate (CS) and hyaluronic acid (HA). GAGs are produced by enzyme-mediated polymerization and can be present as free polysaccharides or associated with glycoproteins. Thus, unlike proteins, changes in GAG composition are less directly linked to gene expression, but instead reflect altered activity of synthetic, metabolizing, and modifying enzyme activity at the tissue level. Given the importance of glycocalyx GAG composition in driving immunological activities in the vasculature and the role these activities play in transplant graft survival, we hypothesize that changes in HS and disaccharides in transplanted grafts may help to identify early or persistent rejection, or even guide new therapeutic approaches to preventing graft rejection.

N-deacetylase-N-sulfotransferase-1 (Ndst1) acts as a central modifying enzyme in HS, catalyzing sulfate conjugation to carbohydrates. Prior work by other researchers has demonstrated that conditional Ndst1 deficiency in endothelial and myeloid precursor cells reduced inflammatory cell invasion^14^, acute antibody-induced nephritis^18^ and allergic airways disease in experimental models^12^. Our own work with aortic allografts, which is a model for chronic transplant vascular inflammation and fibrosis^20,21^, we demonstrated reduced vasculopathy and inflammation at 4 weeks follow-up in *Ndst1*^−/−^ donor aortas implanted in WT BALB/c mice with normal Ndst1 expression (*Ndst1*^+/+^)^19^. Ndst1 deficiency in acute, early rejection in solid organ transplants has not been previously examined, nor have modifications of GAG composition been assessed. Thus the independent functions of glycosaminoglycans (GAG) in donor organs after transplant, and specifically in the endothelial glycocalyx, are incompletely understood^11–14,16–18,22^.

Larger DNA viruses have evolved pan-specific chemokine modulating proteins (CMPs) that inhibit a broad range of chemokines, differing from ligand-specific chemokine antagonists^15,19,23–30^. M-T7 is a 37 kDa Myxomavirus-derived secreted glycoprotein that possesses both broad spectrum, species-independent, C, CC and CXC chemokine inhibitory activity and a rabbit species-specific interferon gamma (IFNγ) inhibitory activity^23–25^. M-T7 point mutations have also been developed with variably modified GAG and chemokine interactions^29^. M-T7 binds the GAG-binding domain of chemokines from multiple species, thereby disrupting diverse classes of chemokine gradients. M-T7 activity was blunted in Ndst1-deficient mouse donor aortic transplants, a model for chronic TAV and vascular injury, but not in CC chemokine receptor deficient aortic allograft transplants, supporting M-T7 interference with chemokine-GAG interactions^19^. Treatment with purified M-T7 protein reduces mononuclear cell invasion and intimal plaque in rodent models of angioplasty injury, as well as in aortic and renal transplants, with improved long term (>100 days) renal allograft survival and reduced scarring. M-T7 treatment effects on acute rejection have not as yet been examined^19,23,26–29^.

We investigate here the effect of isolated donor organ Ndst1 deficiency on early transplant rejection after engrafting *Ndst1*^−/−^ kidneys into WT Ndst1-expressing (*Ndst1*^+/+^) recipient mice. Effects of Ndst1 deficiency on donor solid organ renal allografts were compared to systemic blockade of chemokine-to-GAG interactions by M-T7 in WT renal allografts. Histopathological analysis of acute kidney allograft rejection was correlated with altered tissue immune cell invasion and with gene expression, chemokines, HS and CS GAG, and tissue disaccharides.

## Results

### Ndst1 deficiency in donor renal allografts significantly reduces histopathological markers for early renal allograft rejection

**Figure 1:**
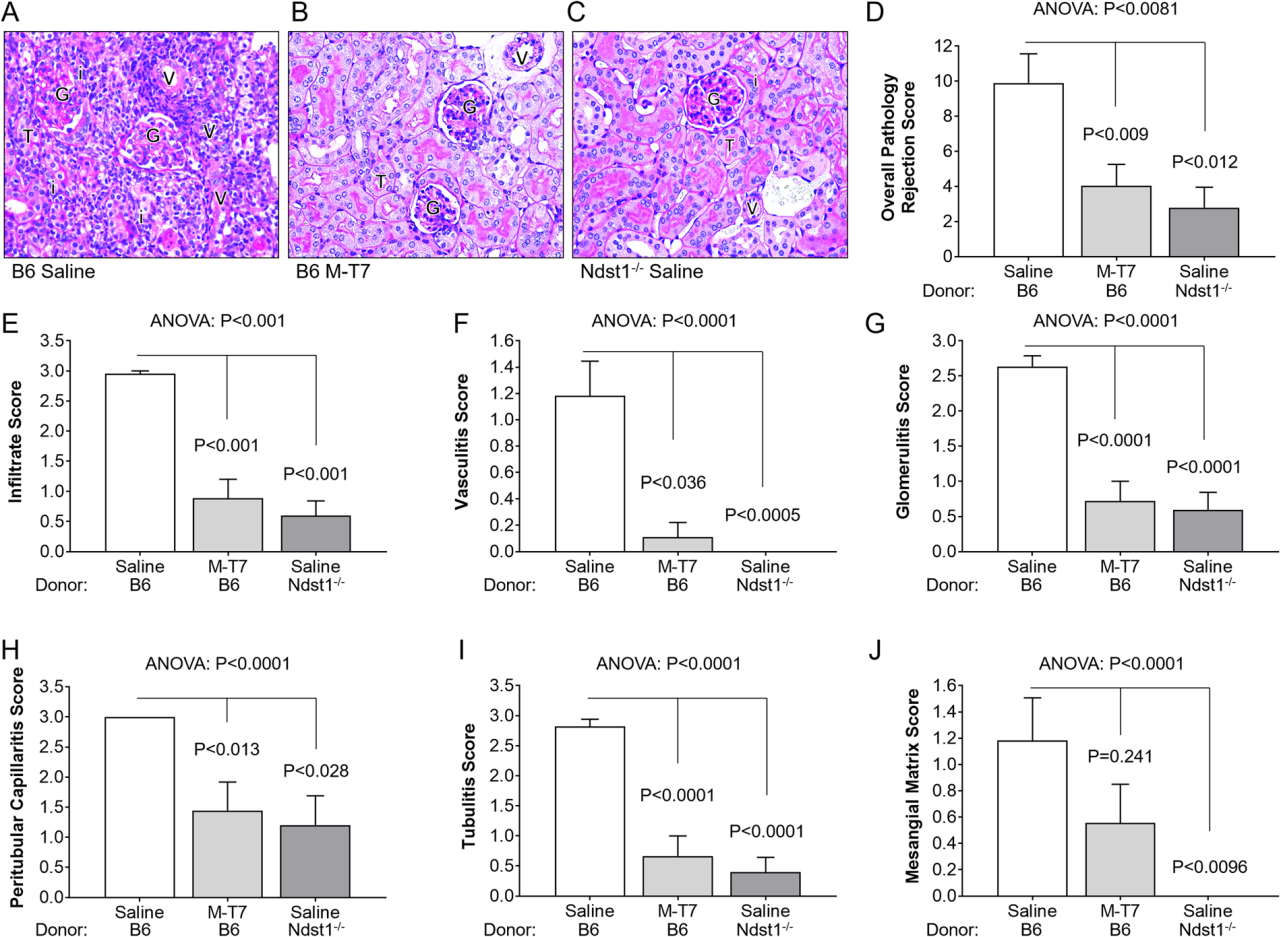
Histopathological analysis of renal allograft sections at 10 days post-transplant follow up for saline treated WT (N = 10 B6 mice) and *Ndst1*^−/−^ (N = 9 mice) donor allografts and M-T7 treated WT allografts (N = 7 mice). WT donor transplants displayed increased histological markers of acute rejection **(A)** which were significantly decreased for M-T7 treated WT allografts **(B)** and *Ndst1*^−/−^ donor transplants **(C)**. Bar graphs illustrate overall pathology rejection score, analyzed for 6 pathological parameters by pathologists blinded to donor organ, demonstrating significantly reduced rejection scores in *Ndst1*^−/−^ and M-T7 treated WT compared to saline-treated WT allografts **(D)**. Bar graphs also demonstrate significant decreases in individual pathologic parameter scores measured in *Ndst1*^−/−^ donors compared to WT donors and for M-T7 treated WT allografts for Infiltrate **(E)**, Vasculitis **(F)**, Glomerulitis **(G)**, Peritubular capillaritis **(H)**, Tubulitis **(I)**, and Mesanginal matrix **(J)**. P-value < 0.05 considered significant. Mag 200X. G, Glomerulus; V, vessel; T, tubule; i, inflammatory infiltrate.

**Figure 2:**
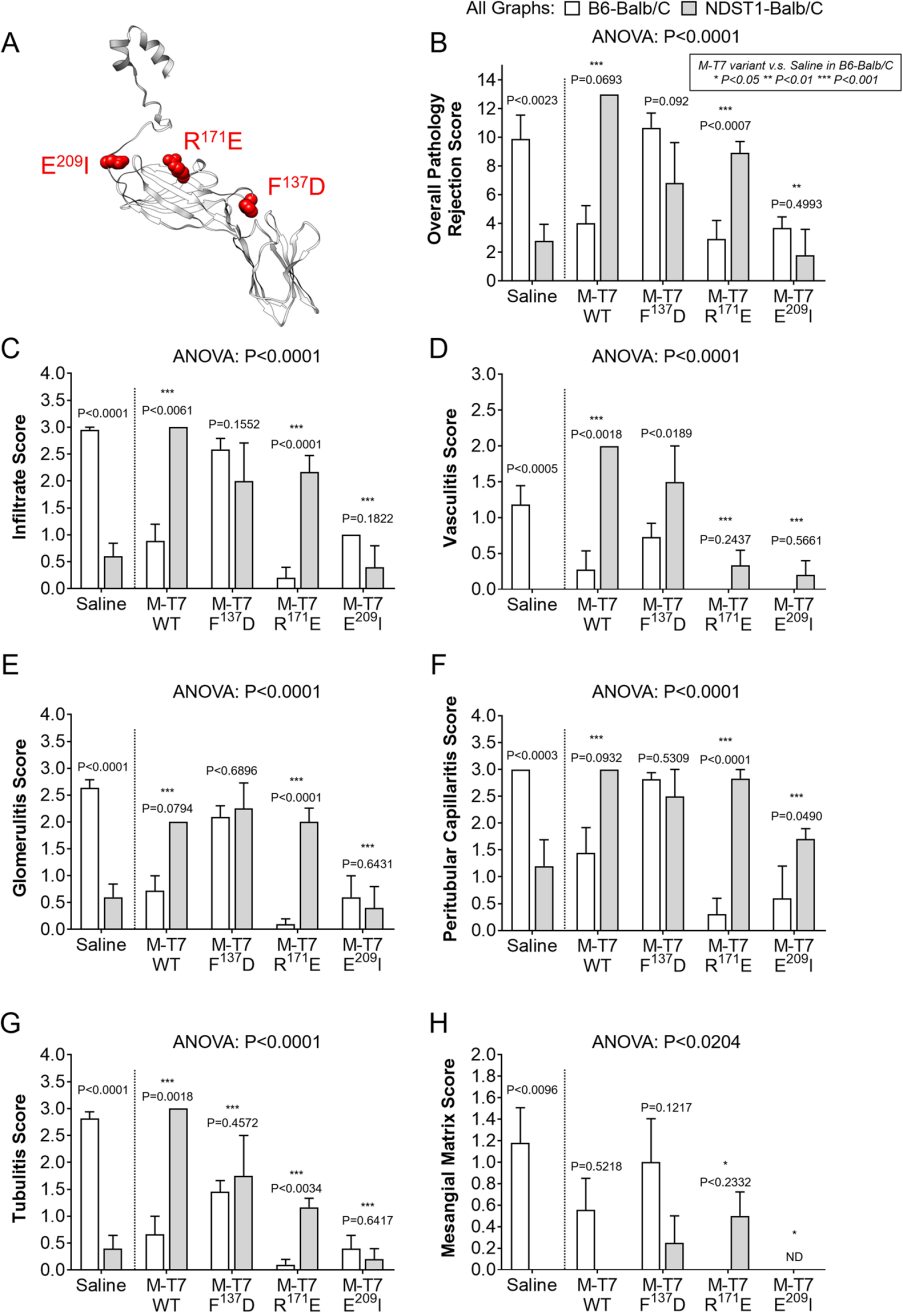
M-T7 variants in wildtype C57Bl/6 (B6, WT) or *Ndst1*^−/−^ transplants into Balb/C mice. SWISS Model visualization of M-T7 indicating the location of the E^209^I, R^171^E and F^137^D point mutations **(A)**. Bar graphs indicating Overall Pathology of Rejection **(B)**, Infiltrate **(C)**, Vasculitis **(D)**, Glomerulitis **(E)**, Peritubular Capillaritis **(F)**, Tubulitis **(G)**, and Mesangial Matrix **(H)** scores for saline treated or M-T7 variant treated wildtype C57Bl/6 (white bars) or *Ndst1*^−/−^ (gray bars) donor tissue. Numerical P-values above bars represent comparisons between wildtype and *Ndst1*^−/−^ grafts for a specific treatment (adjacent bars). Asterisks indicate statistical comparison between saline and M-T7 or M-T7 variant for wildtype donor tissue only (white bars only) with *P < 0.05, **P < 0.01 and ***P < 0.001.

As an initial assessment, the effects of conditional Tek/Tie2 promoter-driven Ndst1 deficiency in donor renal allografts on early rejection was examined in engrafted mice. Histopathological markers for early rejection in grafts with Ndst1 deficiency were compared to wildtype donor allografts, both treated with saline, at 10 days follow up (Fig. 1A,C,D–J). No other immune modulators were given^19,31–33^. Histopathologic sections were read by pathologists blinded to donor mouse strain according to standard guidelines (WC, DW)^34^. C57Bl/6 (B6) renal allografts (wildtype; WT) implanted into BALB/c mice had an expected, marked increase in histological markers of early transplant rejection (Fig. 1A,D). Donor renal allografts from *Ndst1*^−/−^ mice (on C57Bl/6 background) had significantly reduced overall pathology scores for early rejection when transplanted into BALB/c mice (Fig. 1C,D; P < 0.012) as well as significantly lower scores for cell infiltrates (Fig. 1E; P < 0.001), vasculitis (Fig. 2F; P < 0.0005), glomerulitis (Fig. 1G; P < 0.0001), peritubular capillaritis (Fig. 1H; P < 0.028), tubulitis (Fig. 1I; P < 0.0001), and mesangial matrix (Fig. 1J; P < 0.0096) when compared to saline-treated WT/B6 control grafts (Fig. 1D–J).

In view of the fact that the graft recipients have normal Ndst1 expression (*Ndst1*^+/+^), these findings indicate that deficiency of Ndst1 enzyme, specifically in the donor organ endothelial glycocalyx, reduced early allograft and vascular inflammation and rejection.

### M-T7 treatment significantly reduces histopathological markers for renal allograft rejection

Chemokines interact with both GAGs and cell receptors and are reported to alter transplant rejection, with potential effect on donor organ GAG and chemokine interactions during immune responses. Therefore, treatment with the broad-spectrum chemokine-GAG inhibitor M-T7 was examined in WT renal allograft transplants for comparison to *Ndst1*^−/−^ donor allografts. Independent, blinded pathological analysis demonstrated significant reductions in histological markers of early allograft rejection at 10 days follow up with M-T7 treatment (10 daily doses, 100 ng/gm body weight) (Fig. 1B,D–J) in WT donor kidney transplants when compared to WT donor allografts treated with saline control (Fig. 1A). Changes produced by M-T7 were comparable to those seen with saline treatment of *Ndst1*^−/−^ donor allografts implanted into BALB/c recipient mice. M-T7 reduced overall pathology scores for early rejection (Fig. 1D; P < 0.009) with independent reductions in cell infiltrates (Fig. 1E; P < 0.001), vasculitis (Fig. 1F; P < 0.036), glomerulitis (Fig. 1G; P < 0.0001), peritubular capillaritis (Fig. 1H; P < 0.013), and tubulitis (Fig. 1I; P < 0.0001). M-T7 did not reduce the score for mesangial matrix (Fig. 1J; P = 0.241), although treatment did indicate a trend toward reduction.

### M-T7 and M-T7 point mutant treatment have variable efficacy in WT and Ndst1^−/−^ allografts

M-T7-mediated reductions in markers of inflammation and early rejection were lost in *Ndst1*^−/−^ donor transplants (Fig. 2, N = 32 mice). This is consistent with interference with the known M-T7 mediated inhibition of chemokine to GAG binding. However, the independent beneficial effects of both Ndst1 deficiency and M-T7 treatment on reducing inflammation and rejection in renal allografts were lost in combination (i.e., M-T7 treatment in *Ndst1*^−/−^ donor organs) when compared to WT allografts (Fig. 2B–H).

To further define interactions between M-T7, HS-GAG and chemokines, three M-T7 point mutations, with previously characterized variations in chemokine and GAG binding^29^ (Fig. 2A), were assessed for altered effects on rejection in WT C57Bl/6 and also *Ndst1*^−/−^ renal allograft transplant into BALB/c mice. Treatment with the M-T7 point mutations, F^137^D, R^171^E and E^209^I displayed differing inhibitory activities after transplant of C57Bl/6 WT donor kidneys into Balb/C mice (Fig. 2B–H). R^171^E (P < 0.001) and E^209^I (P < 0.01) retained significant inhibitory function in donor WT renal allografts, whereas F^137^D (P = 0.1774) no longer blocked early rejection (Fig. 2B–H).

R^171^E had no inhibitory activity in *Ndst1*^−/−^ donor transplants, as was seen for M-T7 (Fig. 2). R^171^E therefore had minimal differences from the native M-T7 inhibitory activity in this model. Conversely, E^209^I retained inhibitory activity in both WT C57Bl/6 and *Ndst1*^−/−^ donor renal allograft implants at 10 days follow up suggesting that E^209^I-mediated blockade of early signs of rejection is independent of Ndst1 or HS-GAG mediated chemokine interactions. F^137^D was inactive in both WT and in *Ndst1*^−/−^ allografts. In prior work, AlphaScreen assays for R^171^E and E^209^I demonstrated reduced binding to the chemokine RANTES when compared to M-T7 and F^137^D^29^. While all three tested point mutations had reduced RANTES binding *in vitro*, this effect was reduced for R^171^E and E^209^I. E^209^I had the smallest change in binding in the presence of heparin, suggesting that E^209^I may be less affected by heparin interaction with the chemokine-RANTES binding. Both R^171^E and E^209^I retained inhibitory activity for PMA-activated THP-1 cell migration *in vitro*, while F^137^D did not. There were also variations in independent histopathology findings for each mutant. F^137^D had an increase in vasculitis score (Fig. 2D) in Ndst1 deficient mice while E^209^I had a greater reduction in peritubular capillaritis in *Ndst1*^−/−^ allografts (Fig. 2F).

### Reduced early rejection is associated with modified macrophage and T cell invasion

**Figure 3:**
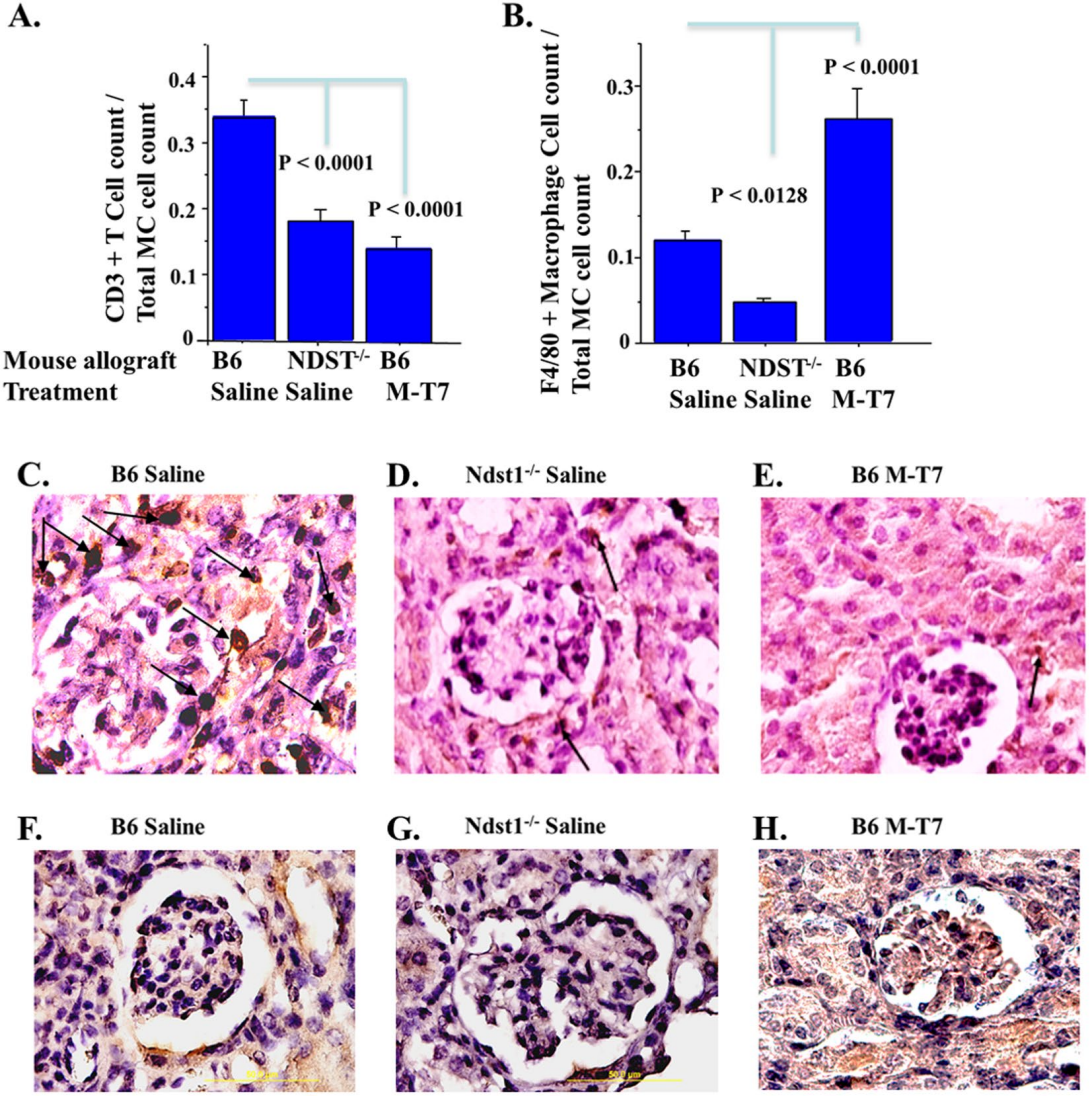
Immunohistological analysis of M-T7 treated WT and saline treated *Ndst1*^−/−^ donor renal allografts at 10 days post-transplant (N = 26 mice). Mean numbers of positively stained cells in 3 high power fields (HPF) are presented as bar graphs demonstrate significant decreases in CD3+ T cell infiltrates for saline treated *Ndst1*^−/−^ donors and for M-T7 treated WT donors **(A)**. F4/80 stained monocytes were significantly decreased in saline treated *Ndst1*^−/−^ donors but significantly increased in M-T7 treated WT donors **(B)**. Representative CD3+ micrographs (100X) for immunohistochemical CD3+ stained grafts, saline treated WT **(C)**, saline treated *Ndst1*^−/−^ **(D)** and M-T7 treated WT **(E)**. Immunohistochemical F4/80 stained graft micrographs for saline-treated WT **(F)**, saline treated *Ndst1*^−/−^ **(G)** and M-T7 treated WT **(H)**. Arrows indicate positively strained cells. P-value < 0.05 considered significant.

Selective changes in macrophage and T cell tissue invasion were assessed in *Ndst1*^−/−^ donor grafts and in M-T7 treated WT donor grafts with comparison to saline treated WT graft controls. Immunohistochemical analysis (Fig. 3) of *Ndst1*^−/−^ allografts as well as M-T7 treated WT allografts demonstrated significant reductions in CD3+ T cells (Fig. 3A,C–E). In contrast F4/80 macrophage cell counts were reduced for saline treated *Ndst1*^−/−^ allografts, but increased in M-T7 treated WT donors (Fig. 3B,F–H).

### Reduced HS and chemokine immunoreactivity is associated with reduced rejection

**Figure 4:**
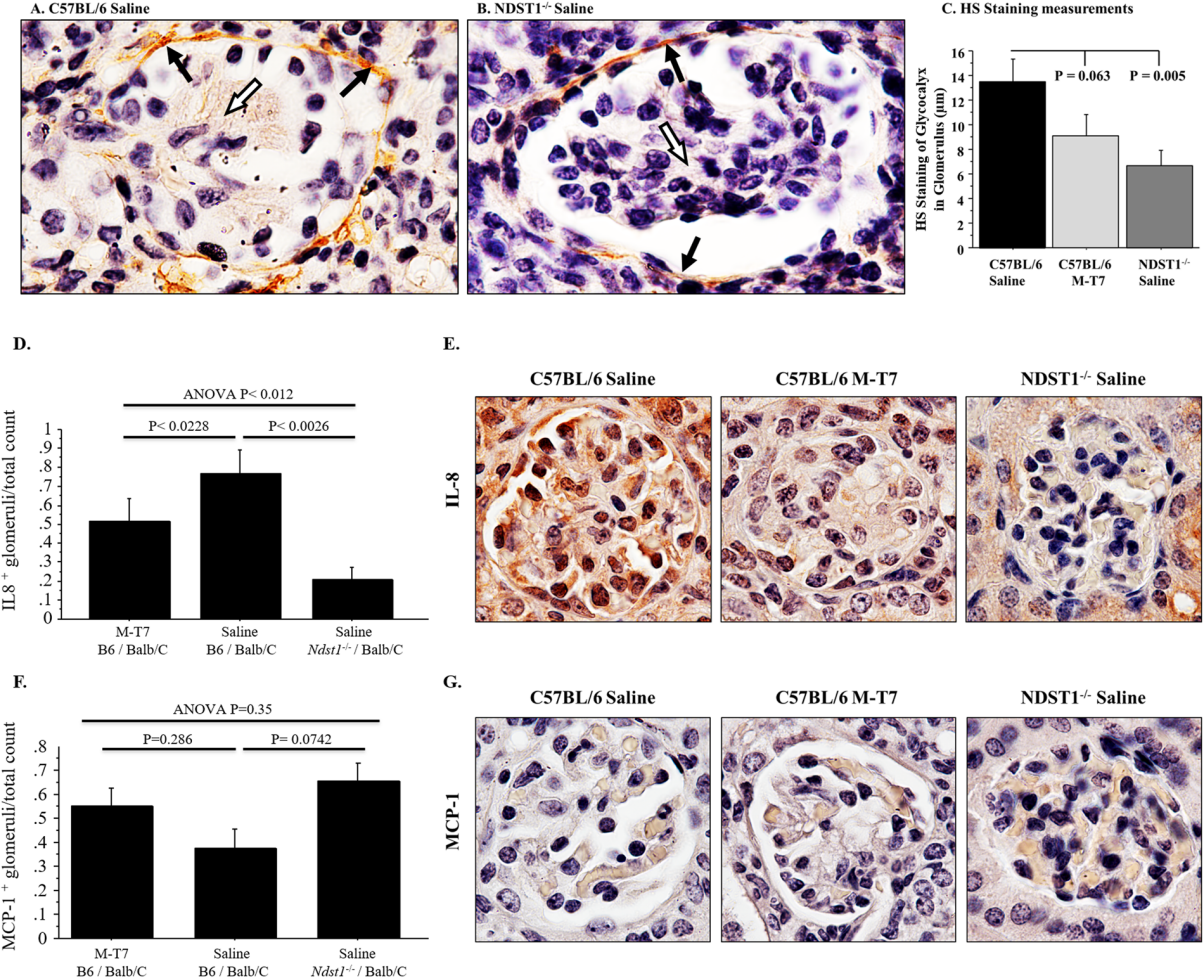
Immunohistochemical analysis of HS and chemokines. **(A)** Glomerular HS staining in saline treated WT allograft. **(B)** Reduced glomerular HS staining in *Ndst1*^−/−^ allografts. **(C)** Bar graph demonstrates significant decrease in HS staining in saline-treated *Ndst1*^−/−^ with only a trend in M-T7-treated WT renal allografts. **(D)** Bar graph demonstrates reduced IL-8 staining in *Ndst1*^−/−^ allographs and in M-T7 treated WT allografts or Saline treated *Ndst1*^−/−^ allografts. **(E)** IL-8 staining in WT allografts compared to *Ndst1*^−/−^ or M-T7 treated allograft glomeruli. **(F)** Bar graph demonstrating no significant decrease in MCP-1 staining in *Ndst1*^−/−^ or with M-T7 treatment of WT renal allografts. **(G)** MCP-1 staining in WT allografts compared to *Ndst1*^−/−^ or M-T7 treated allograft glomeruli. Mag 100X.

Transplanted sections were examined using immunohistochemical staining for HS and chemokines (Fig. 4). Glomerular HS staining in saline-treated *Ndst1*^−/−^ allografts was reduced when compared to saline treated WT transplants (P = 0.005; Fig. 4A–C), consistent with prior reports on *Ndst1*^−/−^ tissue samples. M-T7 treatment demonstrated only a trend toward reduced HS staining, (P = 0.063; Fig. 4C). When chemokine content was assessed, CXC IL-8 staining was significantly reduced in *Ndst1*^−/−^ grafts and in M-T7 treated WT grafts (ANOVA P < 0.012; Fig. 4D,E). CC chemokine MCP-1 was not significantly reduced in either *Ndst1*^−/−^ grafts or in M-T7 treated WT allografts (Fig. 4F,G).

### Altered gene expression in Ndst1^−/−^ allografts and after M-T7 treatment in WT allografts

**Figure 5:**
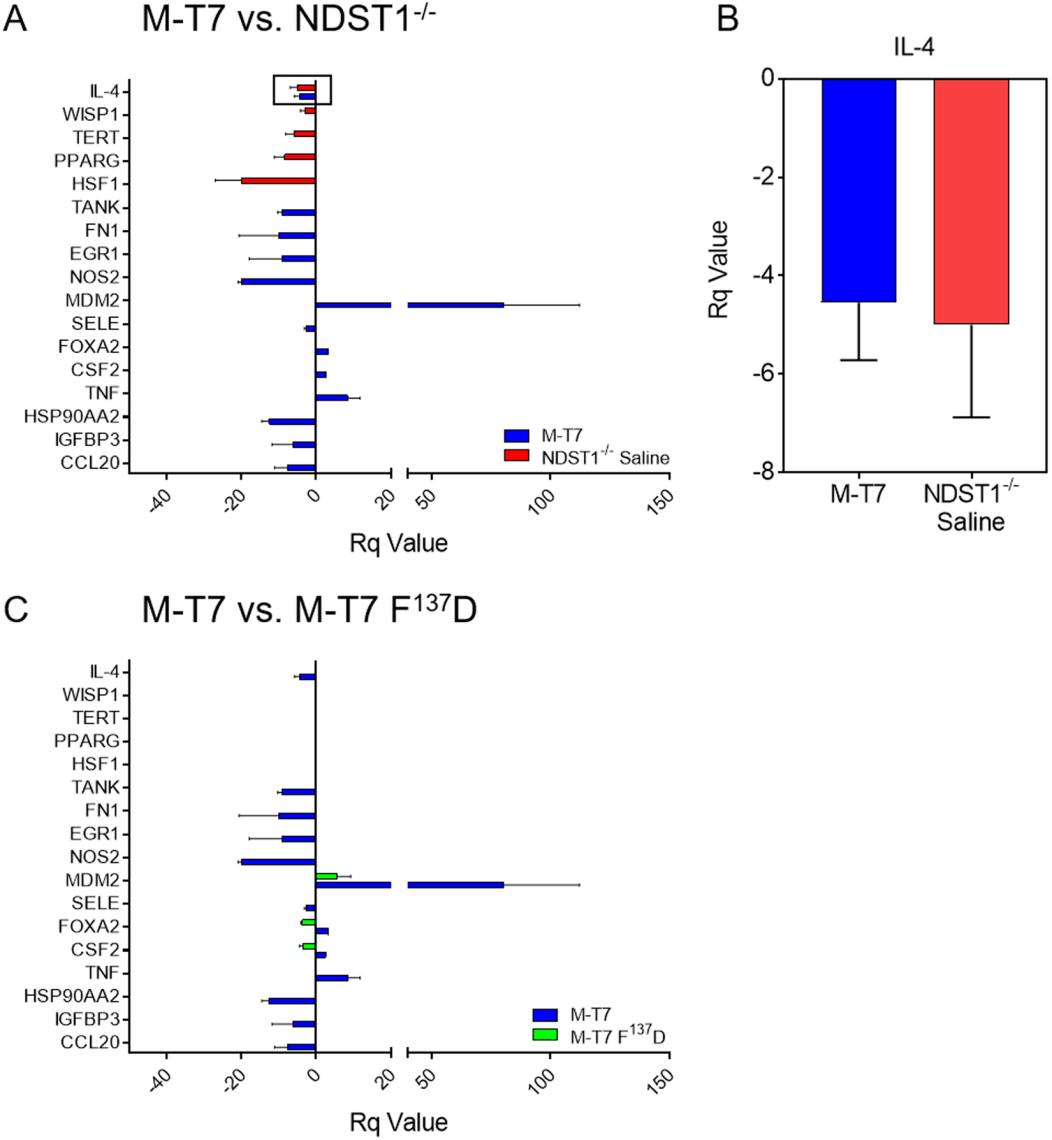
Gene expression changes. Bar graphs illustrating significant changes in gene expression on PCR array analysis of renal allografts sections at 10 days post-transplant (N = 36 mice). **(A)** Comparison of gene expression changes for saline treated *Ndst1*^−/−^ grafts with M-T7 treated WT grafts with comparison to saline treated WT grafts (baseline). **(B)** IL-4 demonstrated significant decrease for both saline treated *Ndst1*^−/−^ (red) and M-T7 treated WT renal allografts (blue). **(C)** Comparison of M-T7 and F^137^D-treated WT grafts with saline-treated WT grafts (baseline).

Changes in gene expression in inflammatory and apoptotic pathways were measured in transplanted organs (Fig. 5). Significantly altered expression was detected for a subset of genes in signaling pathways as detected by qPCR analysis. M-T7-treated C57Bl/6 WT and *Ndst1*^−/−^ donor tissue (both showing reduced rejection) was compared to saline-treated C57Bl/6 WT donors (Fig. 5A). Changes due to treatment with M-T7 and F^137^D mutant (no reduction in rejection) versus saline-treated C57Bl/6 WT donors was also compared (Fig. 5C). Among the detected gene expression changes, Interleukin 4 (IL-4) was significantly decreased for both *Ndst1*^−/−^ and M-T7 treated WT grafts at 10 days follow up (Fig. 5A,B) versus saline-treated WT allografts. Heat shock transcription factor 1 (HSF1), Peroxisome proliferator-activated receptor gamma (PPARG), Telomerase reverse transcriptase (TERT), and WNT1 inducible signaling pathway protein 1 (WISP1) were significantly down-regulated in *Ndst1*^−/−^ grafts, but not M-T7 treated WT allografts. MDM2, CSF2, FOXA2, and TNF were significantly increased for M-T7 treated grafts, but not *Ndst1*^−/−^ grafts. Whereas Nitric oxide synthase 2 (NOS2), TRAF family member-associated NFκB activator (TANK), Early growth response 1 (EGR1), Fibronectin 1 (FN1), CC chemokine CCL20, Heat shock protein 90AA2 (HSP90AA2), IGFBP3, Selectin E (SELE) were decreased in M-T7 treated WT allografts (Fig. 5A).

Specific gene expression changes were all within the NFκB and JAK/STAT pathways, but with changes primarily selective for either Ndst1-deficient grafts or M-T7 treatment in WT grafts. In the NFκB pathway, CCL20 was reduced by M-T7. In the JAK/STAT pathway, Interleukin-4 (IL-4) was significantly reduced in *Ndst1*^−/−^ grafts with saline treatment or in WT allografts with M-T7 treatment. NOS2 was also significantly reduced with M-T7 treatments in WT allografts. Although Murine double minute 2 (MDM2), a p53 regulator, was markedly increased by M-T7, this gene was also increased with F^137^D (Fig. 5C) treatment, which does not reduce rejection, suggesting a poor correlation with reduced rejection.

In summary, a series of genes in inflammatory signaling pathways demonstrated altered expression in grafts with reduced rejection. Significantly reduced IL-4 gene expression was detected for both *Ndst1*^−/−^ allografts and for M-T7 treated WT allografts (Fig. 5B). Significant changes for other genes differed in *Ndst1*^−/−^ grafts when compared to M-T7 treatment in WT grafts, suggesting differing targets.

### Altered HS and CS disaccharide content is detected in renal allografts with reduced rejection

Altered GAG content and metabolism was also examined for correlations with graft rejection. HS and CS disaccharide content and sulfation were measured in isolates from *Ndst1*^−/−^ allografts and from M-T7 or saline-treated WT allografts. Kidney samples vary in weight and thus disaccharides were normalized to total HS or CS content or specimen weight, providing fractional disaccharide content (percentage weight). Total 2-*O*, 6-*O* and N-sulfation in disaccharides were also calculated.

**Figure 6:**
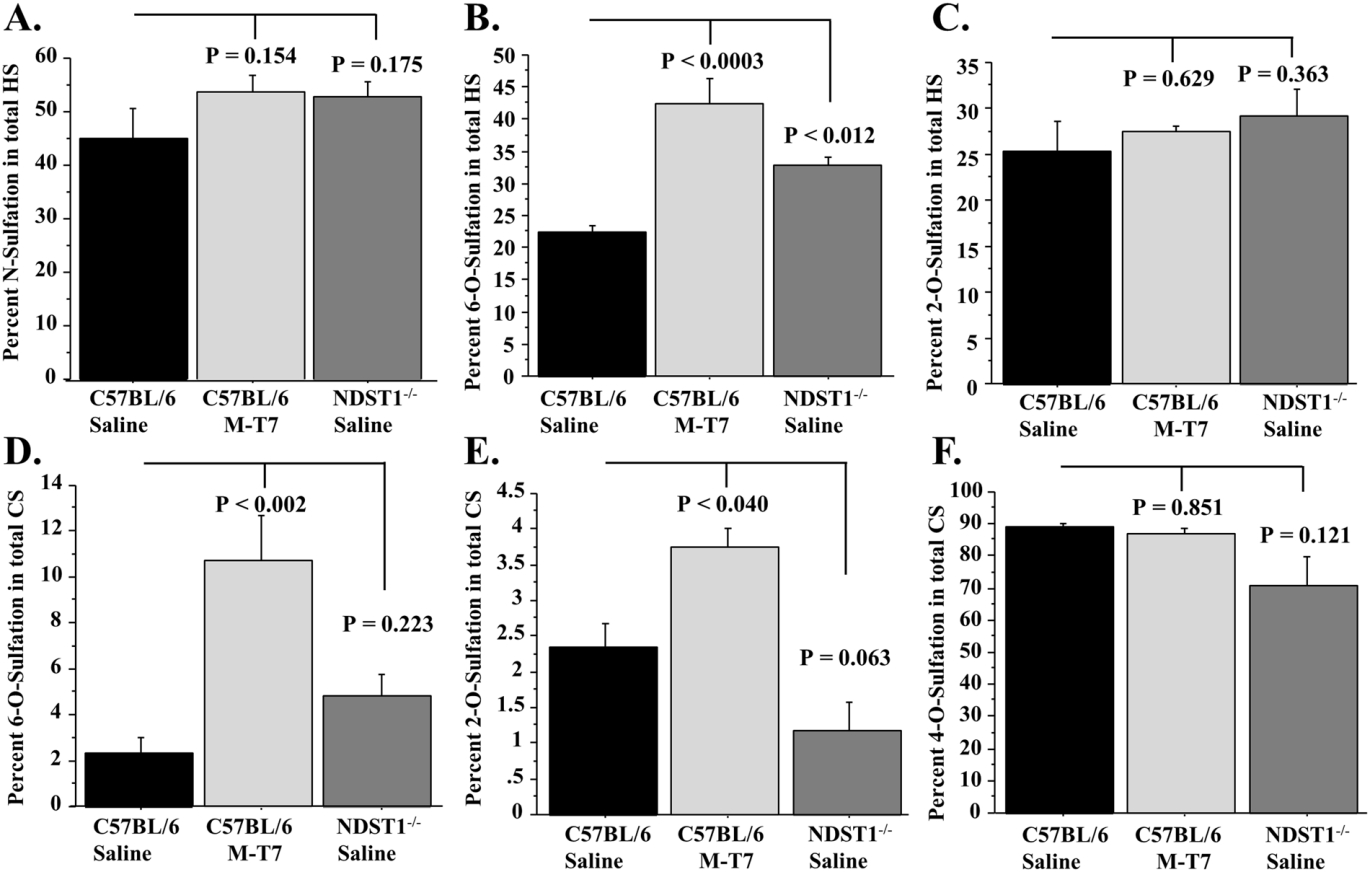
Bar graphs demonstrating total N- and *O*- sulfation changes in disaccharide content, measured as percent weight disaccharides HS **(A–C)** or CS **(D–F)** from saline treated *Ndst1*^−/−^ and saline or M-T7 treated WT mouse kidneys after 10 days treatment; Total HS extract N sulfation **(A)**, Total HS extract 6-*O* sulfation **(B)**, Total HS extract 2-*O* sulfation **(C)**, Total CS extract 6-*O* sulfation **(D)**, Total CS extract 2-*O* sulfation **(E)** and Total CS extract 4–0 sulfation **(F)**.

Overall N-sulfation (sum of D0S0, D0S6, D2S0 and D2S6 content), 6-*O*-sulfation (sum of D0A6, D0S6, D2A6 and D2S6) and 2-*O*-sulfation (sum of D2A0, D2S0, D2A6 and D2S6) as a percentage of total HS was calculated (Fig. 6). Overall N-sulfation in HS extracts was not altered (Fig. 6A). 6-O-sulfation in HS was significantly and unexpectedly increased in M-T7 treated WT transplants and saline treated *Ndst1*^−/−^ transplants compared to controls (Fig. 6B; P < 0.0003 and 0.012, respectively). M-T7 also significantly increased percent 6-*O*-sulfation and 2-*O*-sulfation of CS, whereas *Ndst1*^−/−^ donors did not show an increase when compared to WT saline treatment (Fig. 6D–F).

**Figure 7:**
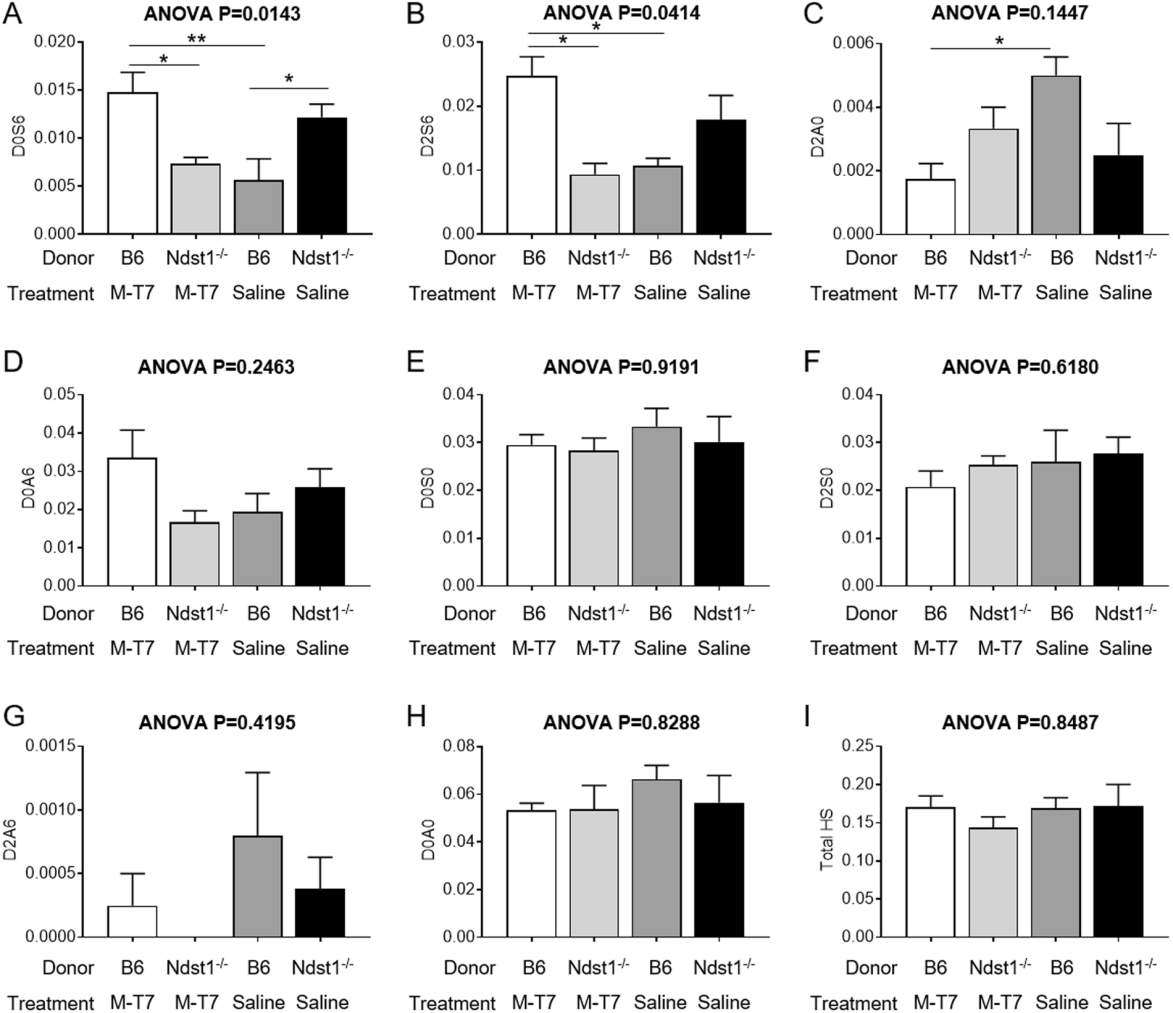
Bar graphs demonstrating changes in disaccharide content, measured as weight per weight fractions of total HS extracts, from saline treated *Ndst1*^−/−^ and saline or M-T7 treated WT mouse kidneys after 10 days treatment. HS Disaccharide analysis for D0S6 **(A)**, D2S6 **(B)**, D2A0 **(C)**, D0A6 **(D)**, D0S0 **(E)**, D2S0 **(F)**, D2A6 **(G)**, D0A0 **(H)** and Total HS **(I)**. D0S6 is increased for both saline treated *Ndst1*^−/−^ and M-T7 treated WT kidneys (**A**), but D2S6 is only significantly increased in M-T7 treated WT grafts (**B**). D2A0 is reduced with M-T7 treatment (**C**). Total HS was not significantly altered (**I**) (*P ≤ 0.05).

*Ndst1*^−/−^ kidneys and M-T7 treated WT kidneys additionally had specific changes in percentage weight HS disaccharide, when compared to saline treated WT kidneys (Fig. 7)^35^. The percent weight (µg) of D0S6 was significantly increased in saline treated *Ndst1*^−/−^ (P < 0.022) and in M-T7 treated renal grafts (P < 0.006) when compared to saline treated WT grafts (Fig. 7A; ANOVA P < 0.0143). Increased D2S6 was also detected in M-T7 treated WT (P < 0.013), but with a non-significant, borderline increase in saline treated *Ndst1*^−/−^ grafts (P = 0.168) (ANOVA P < 0.0414; Fig. 7B). D2A0 was borderline reduced (Fig. 7C) with M-T7 treatment (ANOVA P = 0.1447). Other disaccharides, D0A6 (Fig. 7D), D0S0 (Fig. 7E), D2S0 (Fig. 7F), D2A6 (Fig. 7G), and D0A0 (Fig. 7H) were not significantly modified. Comparison of total HS content also did not detect significant changes (Fig. 7I). With M-T7 treatment after *Ndst1*^−/−^ donor transplant, the D0S6 and D2S6 disaccharide content was comparable to saline treated WT grafts, correlating with a loss of anti-rejection activity when combining M-T7 treatment with *Ndst1*^−/−^ allograft transplant (Fig. 7A,B; P < 0.0177 for M-T7 treatment in *Ndst1*^−/−^ grafts when compared to M-T7 treatment in WT grafts). As the method for labeling disaccharides can be complex, an explanatory diagram for HS disaccharide labeling is provided in Supplementary Fig. S1.

**Figure 8:**
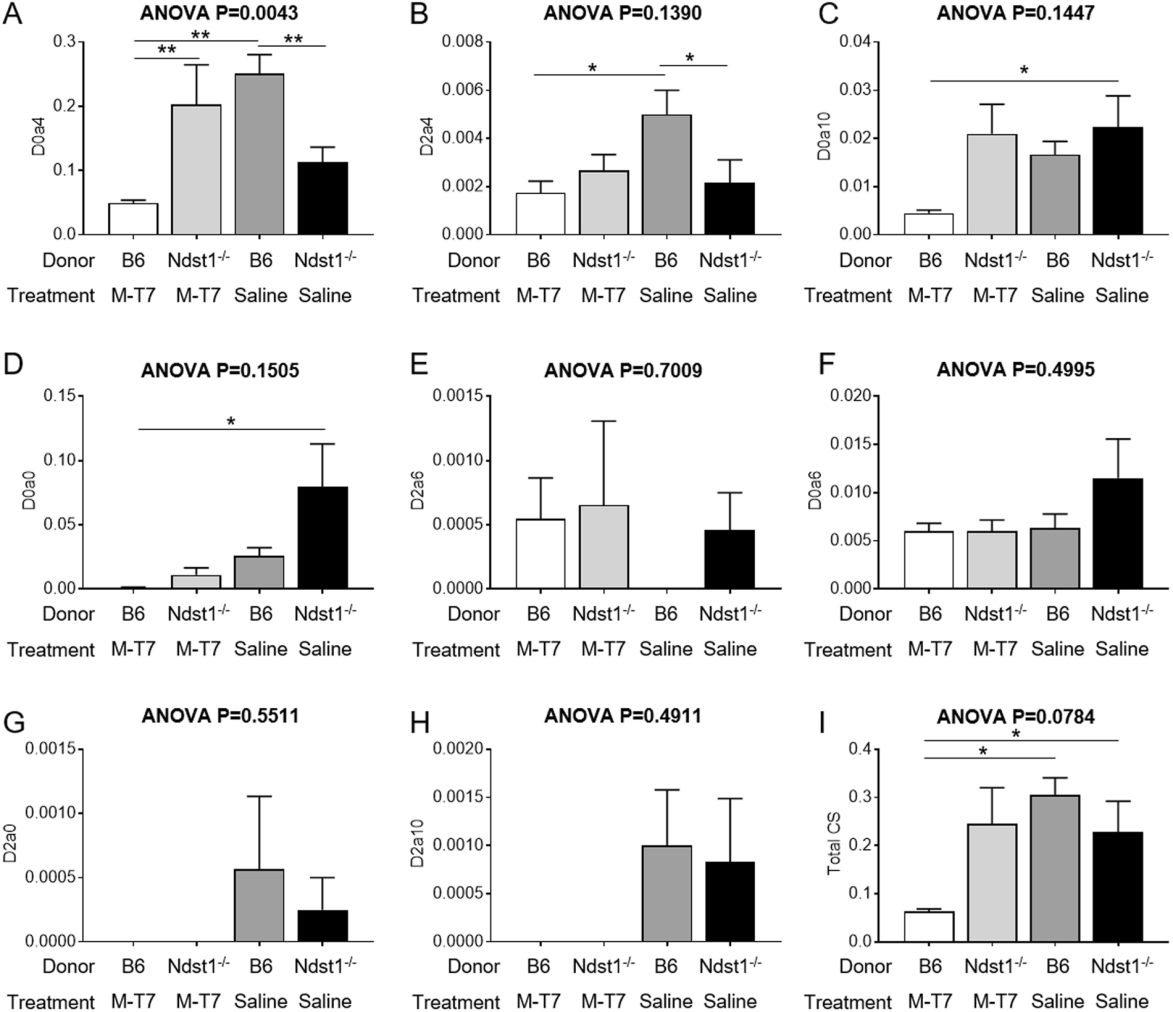
Bar graphs demonstrating changes in disaccharide content, measured as weight per weight fractions of total CS extracts, from saline-treated *Ndst1*^−/−^ and saline or M-T7 treated WT mouse kidneys after 10 days treatment. CS Disaccharide analysis for D0a4 **(A)**, D2a4 **(B)**, D0a10 **(C)**, D0a0 **(D)**, D2a6 **(E)**, D0a6 **(F)**, D2a0 **(G)**, D2a10 **(H)** and Total CS **(I)**. D0a4 (**A**) and D2a4 (**B**) are decreased for both saline treated *Ndst1*^−/−^ and M-T7 treated WT kidneys, but the ANOVA analysis is overall significant only for D0a4. D0a10 is only significantly reduced for *Ndst1*^−/−^ allografts **(C)**. Total CS is reduced for M-T7 treated WT allografts, but again the ANOVA is borderline at P = 0.0784 (**I**) (*P ≤ 0.05).

Significant changes were also observed in CS percent weights or CS disaccharides, although Ndst1 is reportedly selective for HS modification (Fig. 8). D0a4 (Fig. 8A) and D2a4 (Fig. 8B) CS disaccharides were reduced, D0a4 for both *Ndst1*^−/−^ transplants and M-T7 treated WT transplants (ANOVA P < 0.0043) and borderline for D2a4 (ANOVA P = 0.1390). Total CS content (Fig. 8I) was significantly reduced in M-T7 treated renal transplants (P < 0.019), but not in *Ndst1*^−/−^ transplants (ANOVA P = 0.078). Supplemental Figs S2 and S3 provide the HS and CS disaccharide data measurements using the same Y axis scale to allow for comparison of overall changes in content.

Combined changes in measured individual HS and CS disaccharides were correlated with overall pathology rejection scores measured on the same histology sections in *Ndst1*^−/−^ or M-T7 treated WT kidneys by multiple linear regression analysis (for HS disaccharides R = 0.992, R^2^ = 0.984, for CS disaccharides R = 0.974, R^2^ = 0.949).

## Discussion

Early and ongoing activation of inflammatory immune cell responses, also termed innate or acute cellular rejection, are reported to induce ongoing organ damage and to be a significant driving force for late chronic transplant vasculitis, rejection and graft loss^36–44^. Late organ damage is also known to be caused by recurrent antibody-mediated immune rejection. Both antibody-mediated rejection and inflammatory cell responses are reported to contribute approximately fifty percent to ongoing chronic rejection, graft damage and vasculopathy, occurring concomitantly in 25% or more of rejection episodes.

The result of this study on early rejection of renal allograft transplant demonstrates significant reductions in rejection after implant of donor kidneys deficient in Ndst1, the primary sulfotransferase HS-modifying enzyme (Fig. 1). Recipient BALB/c mice in this study have normal (wildtype) Ndst1 expression. Thus, the reduction in early rejection histopathology scores after transplant of *Ndst1*^−/−^ donor organs with saline treatment is unique to the Ndst1*-*deficient donor organ, as no other treatment was given. In prior work, we demonstrated reduced aortic allograft inflammation and vasculopathy at later follow up times (4 weeks) in *Ndst1*^−/−^ donor aortic grafts and after M-T7 treatment in WT aortic allografts^19^. M-T7 also reduced chronic rejection and improved outcome in renal grafts at long term follow up in mice (100 days) and rats (5 months), respectively^19,26^, but was not previously tested for effects on early or acute rejection. The aortic transplant model is considered a model for chronic transplant vasculopathy, more closely representative of chronic arterial inflammation and repair rather than antibody-mediated rejection^43,44^. Thus, Ndst1 deficiency in the donor organ alone reduces both late (i.e., chronic) vasculopathy in aortic allografts and early or acute rejection in renal allografts. Significant and comparable reductions in rejection were also seen after treatment with M-T7, a broad-spectrum chemokine modulating protein that interferes with chemokine-GAG binding (Fig. 1). The capacity to modify rejection by altering GAG composition in the donor allograft may have broad potential for new treatment approaches in transplantation through modifying the donor organ.

While prior work has demonstrated that chemokines have an important role in immune and inflammatory responses in transplants, the role of the endothelial glycocalyx in donor organs has been less extensively studied^1,19,36–43,45^. The capacity of an isolated decrease in Ndst1 expression specifically in the donor organ to significantly reduce early rejection suggests a central role for donor organ HS GAG content in rejection. Further the Ndst1 deficiency is selective for endothelial cells and myeloid precursors and one might predict that changes in donor Ndst1-deficient organs are predominately due to endothelial deficiency rather than myeloid precursors, as observed for thioglycollate-induced peritonitis and allergic contact dermatitis^14^. Because all donor immune cells could not be removed prior to renal transplantation, we cannot exclude the possibility of hematopoetic microchimerism and resident suppression as an involved mechanism in our model^46–48^. Indeed, it is known that LysM (leukocyte)-specific deletion of Ndst1 can affect inflammatory responses^49^. Thus, there is a possibility that resident immune cells in the graft, which may be devoid of Ndst1, may play a role in the maintenance of graft integrity. Wang *et al*. performed control experiments on bone marrow chimeras to note that the predominant inflammation-associated Ndst1 knockout effects in this specific strain of mice are almost exclusively due to knockout in the endothelium^14^. Nevertheless, this remains to be proven in a transplant model and will require further investigation in future studies.

A significant reduction was detected in HS staining and IL-8 CXC chemokine staining with *Ndst1*^−/−^ grafts suggesting that modified HS sulfation can interfere with chemokine-GAG gradient formation. However, M-T7 treatment, while reducing glomerular IL-8 CXC chemokine staining, did not significantly alter HS or CC MCP-1 chemokine staining (Fig. 4; P = 0.063).

We assessed changes in HS and CS content in renal allografts, but unexpectedly found increases in select HS disaccharides suggesting that Ndst1 deficiency reduced HS sulfation in the endothelium, but led to an increase in disaccharides in the whole transplanted organ, potentially due to a response in the graft to the local endothelial changes. There were shared differences in disaccharide content and sulfation for *Ndst1*^−/−^ allografts and M-T7 treated WT allografts. HS disaccharide D0S6 was significantly increased both in *Ndst1*^−/−^ kidneys with saline treatment or in M-T7 treated WT kidneys. With the loss of M-T7 mediated suppression of rejection in treatment of *Ndst1*^−/−^ allografts, there was also a loss of the observed increase in D0S6 and D2S6 disaccharides. Select CS disaccharides, D0a4 and D2a4 were decreased for *Ndst1*^−/−^ grafts and for M-T7 treated WT grafts. Aside from the converging or similar changes in HS and CS disaccharides for *Ndst1*^−/−^ and for M-T7 treatment, our analyses also highlighted numerous diverging GAG composition changes. The increased 6-O sulfation for HS and 2-O sulfation for CS might suggest an inverse reaction in adjacent, non-deficient mouse tissues, *e.g*. around the endothelium, that may react to the Ndst1 deficiency. HS and CS disaccharide analysis was performed on whole *Ndst1*^−/−^ or M-T7 treated WT kidneys and not on endothelial cells alone.

To assess whether an overall change in disaccharide content might correlate with the risk of rejection, MR analysis was performed. A correlation between disaccharide content and rejection scores was detected, again suggesting a correlation between overall GAG content and rejection. Increased 6-*O* heparan sulfation has been previously reported in renal transplant biopsies with increased chronic fibrosis and rejection^41^, thus indicating that altered D0S6 disaccharide content may represent one potential response to allograft rejection, whether protective or damaging^36^. Conversely, sites of low sulfation have been associated with potentially inflammatory endoglycoside heparanase degradation of the glycocalyx and thus increased 6-*O* HS sulfation observed in our study (Fig. 6B) may be protective^50^. Selective changes in disaccharide content may also be specific to individual cells as mast cell responses have demonstrated altered HS content with Ndst1 deficiency^51^. Studies reported by other groups do support a pro-inflammatory function for some GAGs. Heparanase treatment in a donor stem cell transplant model is reported to reduce rejection, improve cell survival^13,18^ and reduce T_H_2 responses and prevent diabetes in mice^36–44^. HS is up-regulated in transplant vasculopathy (TAV) in chronic rejection, as well as in ischemia reperfusion injury^36–38^. Antibodies to selected GAG species are reported to increase rejection^41^. Conversely, low molecular weight heparin infusion reduces scarring as well as transforming growth factor (TGF) and collagen expression after renal obstruction injury in mice. Further work will be required to determine the specific interactions between transplant alone and each treatment in WT and in Ndst1 deficient transplants. Due to the complexity of GAG synthesis and modification, we note that these findings do not demonstrate a direct cause and effect between altered disaccharide composition in donor organs with early rejection, but rather highlight a consistent correlation requiring further study. HS GAGs have multiple functions in addition to chemokine-mediated cell activation and migration and further in-depth analyses will be required to determine the exact mechanism(s) by which acute cell or antibody-mediated rejection is reduced with either Ndst1 deficiency or M-T7 treatment of allografts. We also report here a reduced efficacy for the M-T7 F^137^D point mutation in treating WT kidney grafts, suggesting that the beneficial effect of M-T7 in acute rejection with WT donors is specific to M-T7 mediated inhibition of chemokine binding to GAG alone and not through direct M-T7 interaction with GAG. The F^137^D mutant is predicted to have reduced chemokine interaction when compared to native M-T7 as the hydrophobic region of the structure that interacts with chemokines is disrupted. This supports the hypothesis that M-T7 reduces transplant rejection via inhibition of chemokines. In prior work F^137^D had reduced inhibition of chemokine binding when treated with heparin^29^. However, the finding here for F^137^D, differed from prior findings examining plaque growth in a mouse model of balloon angioplasty injury in hyperlipidemic ApoE^null^ mice, where F^137^D retained some inhibitory activity, but did mirror the loss of inhibition for PMA activation on monocytes *in vitro*^29^. E^209^I was less affected by heparin competition in prior work *in vitro*^29^, and retained activity in *Ndst1*^−/−^ allografts in the current study. These observations suggest the possibility that E^209^I protein functions independent of GAG interactions. This difference in responsiveness for balloon angioplasty injury and solid organ transplant is not unexpected, as mechanisms underlying acute organ transplant rejection differ from those driving plaque growth after simple mechanical balloon angioplasty injury where there is no rejection.

Selective reduction in transplant organ CD3+ T cell invasion correlated with reduced rejection. The individual changes in modification of inflammatory response pathway genes support a key role for modification of innate and acquired immune responses in rejection, both via Ndst1 deficiency and with M-T7 treatment. The reduction in IL-4 gene expression by both approaches, M-T7 treatment in WT allografts and in saline treated *Ndst1*^−/−^ allografts, does suggest again shared or convergent regulatory pathways for *Ndst1*^−/−^ donor grafts and for M-T7 treatment in WT grafts. While the change in MDM2 is large and may represent a significant regulatory step, there is no comparable increase in *Ndst1*^−/−^ kidney and treatment with the inactive F^137^D point mutation also increased this gene, making this change of lesser interest. A transcriptome-wide analysis for genes that correlate with histopathological changes in rejection would be preferable, and will be approached in future analyses^34^.

These findings correlate the reduced rejection observed in saline treated *Ndst1*^−/−^ engrafted mice to altered HS and CS content, potentially via blockade of chemokine interactions. The observed reduction in overall rejection score is very similar to systemic treatment with M-T7, a broad-spectrum inhibitor of chemokine-GAG interactions. However, modulation of other GAG-dependent functions has yet to be examined in this model, and immune modulators other than chemokine-GAG interactions may play key functions. Further work will be necessary to examine other potential HS GAG interactions modified by Ndst1 deficiency or M-T7 treatment.

## Conclusions

In conclusion, reducing endothelial and myeloid precursor cell Ndst1 expression, in donor allograft transplants alone, reduces acute renal allograft rejection, comparable to chemokine inhibition. Changes in donor organ HS disaccharide composition in transplanted organs has potential for diagnosis in detecting early rejection. These findings may also guide new donor-focused approaches to treating transplant organs designed to reduce acute inflammation and prevent chronic allograft damage, which is an unmet therapeutic need.

## Materials and Methods

### Animals

Three strains of mice were used in this study. C57BL6/J (stock #000664) and BALB/c (stock #000651) mice were obtained from JAX Laboratories (Bar Harbor, MN) or the University of Florida Animal Care Services breeding facility, which replenishes stocks from JAX Laboratories. Derivation and characterization of Ndst1^f/f^TekCre^+^ mice (*Ndst1*^−/−^; kindly provided by Dr. Jeffrey D. Esko, Glycobiology Research and Training Center, University of California, San Diego, CA) with floxed Ndst1 conditionally knocked out by Tek/Tie2 endothelial tyrosine kinase promoter-driven Cre in the endothelial and sub-population leukocytes have been previously described^14^. All animals were housed in barrier conditions in vivaria of the University of Florida Animal Care Services. Mice were weaned at 3 weeks, maintained on a 12-hour light–dark cycle and were fed water and standard rodent chow *ad libitum*.

### Surgical protocols - Kidney transplantation

All animal studies complied with University and National Institutes of Health guidelines for the care and use of Laboratory animals and were approved by the University of Florida (UFL) and Arizona State University (ASU) Institutional Animal Care and Use committee (IACUC; UFL IACUC Protocol # 201604234_01; ASU IACUC Protocol #17‐1549R). Renal allograft transplant was performed as previously described (Table 1; 6–10 mice with allograft transplant per donor organ genetic strain and treatment type; Total 80 mice). In brief, the donor kidney is placed in the left flank in the mouse and attached by end-to-side anastomosis between the donor suprarenal aortic cuff and the recipient aorta. Venous anastomosis between donor suprarenal inferior vena cava (IVC) and recipient IVC is performed in the same fashion and the bladder attached, as previously described^19^.

**Table 1:** Mouse Renal Allograft Model.

Donor	Recipient	Treatment	Number mice	Follow up (days)	Survival
C57Bl/6	Balb/C	Saline	10	10	10/10
NDST1^−/−^	Balb/C	Saline	9	9	9/9
C57Bl/6	Balb/C	M-T7 (100 ng/gm)	7	10	7/7
NDST1^−/−^	Balb/C	M-T7 (100 ng/gm)	6	6	6/6
C57Bl/6	Balb/C	R^171^E (100 ng/gm)	10	10	10/10
NDST1^−/−^	Balb/C	R^171^E (100 ng/gm)	6	6	6/6
C57Bl/6	Balb/C	F^137^D (100 ng/gm)	10	10	10/10
NDST1^−/−^	Balb/C	F^137^D (100 ng/gm)	6	6	6/6
C57Bl/6	Balb/C	E^209^I (100 ng/gm)	10	10	10/10
NDST1^−/−^	Balb/C	E^209^I (100 ng/gm)	6	6	6/6
Total numbers of mouse renal allografts			80	80	80/80 (100%)

Ndst1^−/−^ conditional Ndst1 deficiency in endothelial cells and myeloid precursors (NDST1^f/f^ TekCre^+^)

A series of donor renal allografts from either C57Bl/6 wild type (WT) or *Ndst1*^−/−^ were transplanted into BALB/c mice. Mice with WT or *Ndst1*^−/−^ donor allografts were treated with either saline control, M-T7 or individual mutated constructs (M-T7-His_6X_, F^137^D, R^171^E, or E^209^I; 6–10 mice per donor organ strain and per treatment) (Table 1)^29,34,52^. Donor renal allografts were transplanted into BALB/c mice after resection of both kidneys under general anesthetic. No other immune suppressants were given to the mice before or after transplantation, in order to examine isolated early effects of each condition alone on transplant rejection at 10 days follow up^19,31–33^. Treatments were given daily by intraperitoneal (IP) injection at 100 ng/gm/day X 10 days per mouse for each individual protein treatment^19^. Mice were euthanized at 10 days follow up with Euthanyl (Virbac AH Inc., Fort Worth, TX) as previously described^19^. For tissue analyses, renal allografts were divided into 3 sections and each section cut in half; one third fixed in 10% neutral-buffered formalin for histology and the other two thirds cut in half and stored frozen or stored in RNAlater for RNA analysis.

### M-T7 and M-T7 point mutation generation and expression

M-T7 and M-T7 point mutations were expressed and purified as previously described. In brief, M-T7 mutants were generated by mutagenic PCR using M-T7pFastBacDualeGFP as the template^112^. Mutant constructs and wild type M-T7 were transformed into DH10Bac bacteria (Invitrogen.Carlsbad, CA), and blue/white screened on LB + Kan + Tet + Gen + IPTG+ X-gal plates. Bacmids were purified and used to transfect Sf9 insect cells with Cellfectin II (Invitrogen. Carlsbad, California). Baculovirus supernatants were collected to infect insect cells and express the various M-T7 mutant proteins. M-T7 and each of the three mutant constructs were then purified by sequential column purification as previously described^19,29^.

### Histological and immunohistochemical analysis of acute rejection and scarring

Sections of transplanted organs were cut into three 1.5–2 mm equal length cross sections for histology, fixed, paraffin embedded, and cut into 4–5 µm sections (3 sections per transplant specimen, providing 9 sections per allograft). Histology sections were stained with Haematoxylin and Eosin (H & E), Masson’s trichrome, and Periodic acid-Schiff (PAS) as previously described^19,23–29^. All sections were analyzed for changes consistent with acute rejection and vasculitis^34^ by pathologists blinded to the mouse donor allograft implant and to treatment with either saline or M-T7 using Banff diagnostic criteria (DW, WC, BC). Pathology was scored on a scale of 4 and overall pathology score was a summation of independent scores assessed by detection of cellular infiltrate, vasculitis, glomerulitis, peritubular capillaritis, tubulitis, and mesangial matrix.

Renal allografts were assessed by immunohistochemical staining for macrophage and T lymphocyte invasion and for HS with rabbit polyclonal against F4/80 (macrophages; ab100790 from Abcam at 1:50 dilution), rabbit polyclonal against CD3 (T cells; ab5690 from Abcam at 1:50 dilution) and mouse antibody to 10E4 epitope of HS (Clone F58–10E4 from AMSBio at 1:50 dilution) using ABC staining technique, respectively and as previously described^19,21–27,29^. Sections were also stained with goat polyclonal against MCP-1 (CCL-2; AF-479-SP from R&D Systems at 1:200 dilution), and rabbit polyclonal against IL-8 (CXCL-8; orb322984 from Biorbyt at 1:100 dilution) chemokines. Sections were incubated with goat anti-rabbit or anti-mouse or donkey anti-goat secondary antibody, as indicated, and counterstained with Haematoxylin. Mean numbers of positively stained immune cells in three high power fields (100X, HPF) were measured for each graft using the Image Pro MC 6.0 trace application program or cellSens Dimensions v1.15 with an Olympus BX51 microscope imaging system, calibrated to microscope objective, as previously described^19,23–29^. Chemokine staining in 6 representative glomeruli in transplanted kidneys (40X) and the thickness of HS stain on membranes at 2 sites in each of 3 glomeruli was measured in HPF (100X) by an investigator blinded to the mouse strain and treatment. Mean cell count and staining thickness per HPF was calculated.

### RT-PCR array analysis of altered gene expression in renal allografts

One third of each transplanted kidney section was collected in RNAlater (Ambion, Austin, TX) and RNA was isolated using RNeasy Mini kit following the manufacturer’s protocol (Qiagen, Valencia, CA). RNA was reverse transcribed to cDNA using Superscript VILO cDNA Synthesis kit (Invitrogen Corporation, Carlsbad, CA) and Real Time PCR carried out using SYBR Green Core Reagent kit and a 7300 RT-PCR system (Applied Biosystems, Austin, TX). Changes in gene expression were normalized to internal GAPDH control and subsequently to saline treated controls. Primers specific to inflammatory and apoptotic pathways were assayed and are listed in Supplemental Table 1.

### Analysis of GAGs from saline treated Ndst1^−/−^ and WT kidneys, with and without M-T7

Total HS- and CS-GAG content and percent weight disaccharide were measured in transplanted kidneys from WT mice, with and without M-T7 treatment, and in transplanted kidneys from *Ndst1*^−/−^ mice with saline or M-T7 treatment (N = 10, 3–4 mice per strain and treatment group). Researchers were blinded to samples (SA, PA). HS and CS GAG composition were quantified by HPLC^53^. Formalin-fixed, paraffin-embedded samples were extracted as previously described^54^. Samples were incubated in glass tubes in a heating block at 60 °C with 3 mL of xylene, heated for 15 minutes with 2 repeats, and then rinsed with 3 mL of 100% ethanol, followed by 96, 70, 50, and 30% ethanol in water and washed three times with 18 MΩ water. Samples were rehydrated in 1 mL solution of PBS for >30 minutes at RT. Rehydrated tissue was homogenized and defatted in acetone over 48 h with shaking at 4 °C. Samples were dried and suspended in 2 mL of 0.1 M Tris-HCl, pH 8.0, containing 2 mM CaCl_2_, and 1% Triton X-100. Pronase (0.8 mg/mL) was added to the suspension and tissue digested at 50 °C for 24 h with one 24 h repeat. Finally, pronase was inactivated by heating to 100 °C for 15 min. Buffer was then adjusted to 2 mM MgCl_2_, benzonase (100 mU) added, and samples incubated for 2 h at 37 °C. After inactivation of the enzyme (15 min, 100 °C), undigested tissue was removed by centrifugation for 1 h at 4000 *g*.

Sample supernatant was applied to a DEAE-Sepharose – *micro* spin column (Harvard Apparatus), washed with ~10 column volumes (CVs) of loading buffer (~pH 8.0 Tris Buffer) twice, allowed to adhere, washed with ~20 CVs of loading buffer, followed by ~20 CVs of wash buffer (~pH 4.0 Acetate Buffer), and 3 CVs of water. Samples were further cleaned with ~15 CVs of 0.2 M NH_4_HCO_3_. To elute, ~20 CV of 2 M NH_4_HCO_3_ was added to the column and the fractions were collected. Samples were then freeze-dried and dissolved in 100 µL water.

Samples were digested with Heparinases I-III (New England Biolabs) or Chondroitinase ABC (Sigma), producing disaccharides that were separated using SAX-HPLC coupled to post-column fluorescence labeling and detection (Agilent system using a 4.6 × 250 mm Waters Spherisorb analytical column with 5 μm particle size at 25 °C). Peak migration times and areas were calculated compared to known disaccharide standards. Representative chromatograms are provided in Supplemental Fig. 4. HPLC were run with two solvents, Solvent A: 2.5 mM sodium phosphate, pH 3.5 and Solvent B: 2.5 mM sodium phosphate, 1.2 M NaCl, pH 3.5 with gradated change from 97% A and 3% B to 100% B and 0% A (65 min, flow rate 1.0 mL/min). GAG detection was performed by post-column derivatization. Eluents were combined with a 1:1 mixture of 0.25 M NaOH and 1% (w/v) 2-cyanoacetamide pumped at a flow rate of 0.5 mL/min from a binary HPLC pump (SSI Scientific Systems, Inc) and heated to 130 °C in a 10 m reaction coil, before cooling and return into the Agilent’s fluorescence detector (λ_ex_ = 346 nm, λ_em_ = 410 nm). Commercial standard disaccharides (Dextra Laboratories) were used for identification based on elution time, as well as for calibration.

Paraffin-containing formalin from each sample was assessed in parallel as a control. No GAG or disaccharide was detected in these controls. Following isolation the GAGs released with β-elimination (1% w/w sodium borohydride in 2N NaOH) were desalted with a PD-10 column (GE Healthcare), and freeze-dried before disaccharide composition analysis. Peak migration times and areas for disaccharides separated by HPLC were compared to known standards (See example in Supplemental Fig. S4).

### Statistical analysis

Measured change in histopathology scores, incorporating tissue mononuclear cell count, percentage of positively stained cells, PCR array, HS staining, and tissue HS and CS disaccharide content were assessed for statistical significance using analysis of variance (ANOVA) with secondary Fisher’s PLSD or Student’s unpaired, two tailed T-test, as previously described^19,23–29^. Multiple and simple regression analyses were performed to assess correlations between tissue disaccharide content with total histopathologic score for acute rejection^35^.

## Electronic supplementary material

Supplementary Figures, Table
