# Supplementary material for: Selective Deletion of Heparan Sulfotransferase Enzyme, Ndst1, in Donor Endothelial and Myeloid Precursor Cells Significantly Decreases Acute Allograft Rejection
Source: Sci Rep. 2018 Sep 7;8:13433. doi: 10.1038/s41598-018-31779-7 (PMC6128922; doi:10.1038/s41598-018-31779-7)
Supplement: Supplementary file 1 — Supplementary Figures, Table [file 41598_2018_31779_MOESM1_ESM.pdf]

SUPPLEMENTARY MATERIAL FOR

Selective Deletion of Heparan Sulfotransferase Enzyme, Ndst1, in Donor Endothelial and Myeloid Precursor Cells Significantly Decreases Acute Allograft Rejection

Hao Chen, MD, PhD[*a], Sriram Ambadapadi, PhD[*b,c,d], Dara Wakefield, MD*[e], Meeyong Bartee, PhD[b], Jordan R. Yaron PhD[d], Liqiang Zhang, PhD[d], Stephanie A. Archer-Hartmann, PhD[f], Parastoo Azadi, PhD[f], Michelle Burgin[d], Chad Borges, PhD[d], Donghang Zheng, MD, PhD[b], Kevin Ergle, MD[b], Vishnu Muppala, BSc[b], Sufi Morshed, PhD[b], Kenneth Rand, MD[e], William Clapp, MD[e], Amanda Proudfoot, PhD[g], Alexandra Lucas, MD†[,b,c,d]

*to be considered co-first authors

[a]The Department of Tumor Surgery, Second Hospital of Lanzhou University, Lanzhou, China

[b]Divisions of Cardiovascular Medicine and Rheumatology, Department of Medicine, University of Florida, Gainesville, FL, USA

[c]Department of Molecular Genetics and Microbiology, College of Medicine, University of Florida, Gainesville, FL, USA

[d]Center for Personalized Diagnostics, and the Center of Immunotherapy, Vaccines and Virotherapy, The Biodesign Institute, Arizona State University, Tempe, AZ, USA

[e]Department of Pathology, University of Florida, Gainesville, FL, USA

[f]Complex Carbohydrate Research Center, University of Georgia, Athens, GA, USA

[g]Merck Serono, Darmstadt, Germany

Supplemental Figure Legends

Supplementary Figure S1. Labeling schematic for the HS disaccharide structure code.

Supplementary Figure S2. Bar graphs demonstrating changes in disaccharide content, measured as weight per weight fractions of total HS extracts, from saline treated *Ndst1*$^{-/-}$ and saline or M-T7 treated WT mouse kidneys after 10 days treatment. All measurements are presented with the same Y axis scale. for D0S6 **(A)**, D2S6 **(B)**, D2A0 **(C)**, D0A6 **(D)**, D0S0 **(E)**, D2S0 **(F)**, D2A6 **(G)**, D0A0 **(H)** and Total HS **(I)**. D0S6 is increased for both saline treated *Ndst1*$^{-/-}$ and M-T7 treated WT kidneys (**A**), but D2S6 is only significantly increased in M-T7 treated WT grafts (**B**). D2A0 is reduced with M-T7 treatment (**C**). Total HS was not significantly altered (**I**) (*P≤0.05).

Supplemental Figure S3.

Bar graphs demonstrating changes in disaccharide content, measured as weight per weight fractions of total CS extracts, from saline treated *Ndst1*$^{-/-}$ and saline or M-T7 treated WT mouse kidneys after 10 days treatment. CS Disaccharide analysis for D0a4 **(A)**, D2a4 **(B)**, D0a10 **(C)**, D0a0 **(D)**, D2a6 **(E)**, D0a6 **(F)**, D2a0 **(G)**, D2a10 **(H)** and Total CS **(I)**. D0a4 (**A**) and D2a4 (**B**) are decreased for both saline treated *Ndst1*$^{-/-}$ and M-T7 treated WT kidneys, but the ANOVA analysis is overall significant only for D0a4. D0a10 is only significantly reduced for *Ndst1*$^{-/-}$ allografts **(C)**. Total CS is reduced for M-T7 treated WT allografts, but again the ANOVA is borderline at P=0.0784 (**I**) (*P≤0.05).

Supplementary Figure S4: Representative HPLC chromatograms for disaccharide analyses.

Supplementary Table 1 – Gene list in gene expression array.

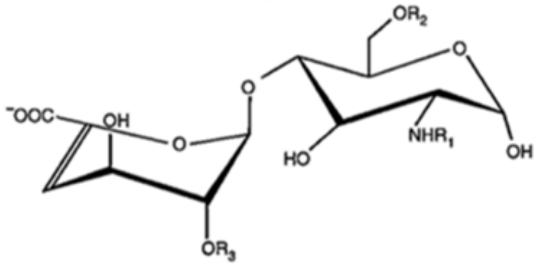

Disaccharide	R1	R2	R3
D0A0	Ac[1]	H[2]	H
D0A6	Ac	Sulf[3]	H
D2A0	Ac	H	Sulf
D2A6	Ac	Sulf	Sulf
D0S0	Sulf	H	H
D0S6	Sulf	Sulf	H
D2S0	Sulf	H	Sulf
D2S6	Sulf	Sulf	Sulf

[1] – acetyl group, [2] – hydrogen, [3] – sulfate group

The four-character descriptor for disaccharides according to disaccharide structure code[1]:
D = $\Delta^{4,5}$ –unsaturated uronic acid
0 = no sulfation at the O-group of the $\Delta^{4,5}$ –unsaturated uronic acid (this could be 0, 2, 3, or 6 signifying no sulfation, 2-O-, 3-O- and 6-O-suafation, respectively)
A = N-acetylglucosamine (it becomes S, if glucosamine is N-sulfated)
0 = no sulfation at the O-group on the glucosamine (this could be 0, 3, 4 or 6 signifying no sulfation 3-O-, 4-O and 6-O sulfation, respectively)

Supplementary Figure 1. Labeling schematic for the HS disaccharide structure code.

Supplemental Figure S2

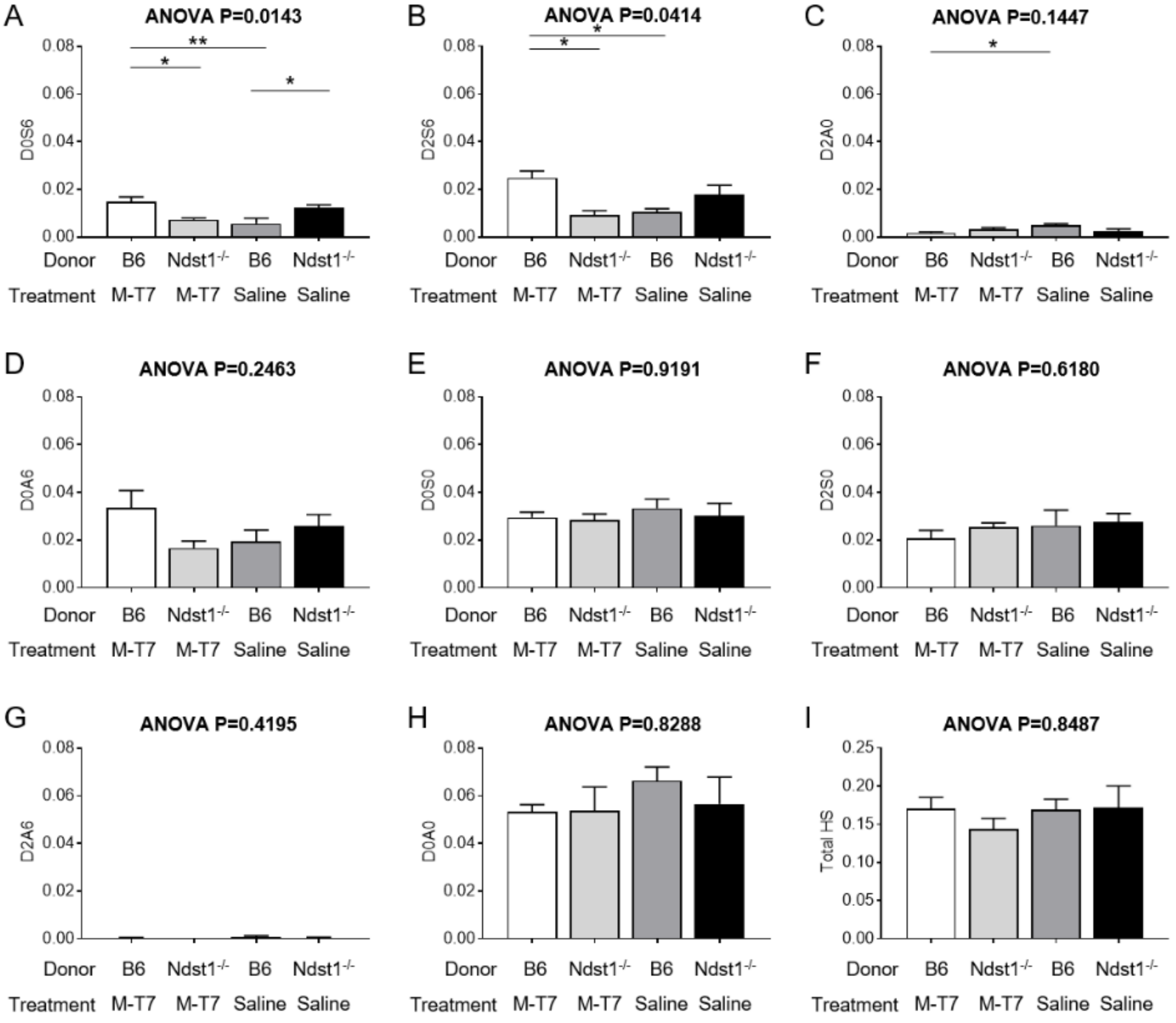

Supplemental Figure S3

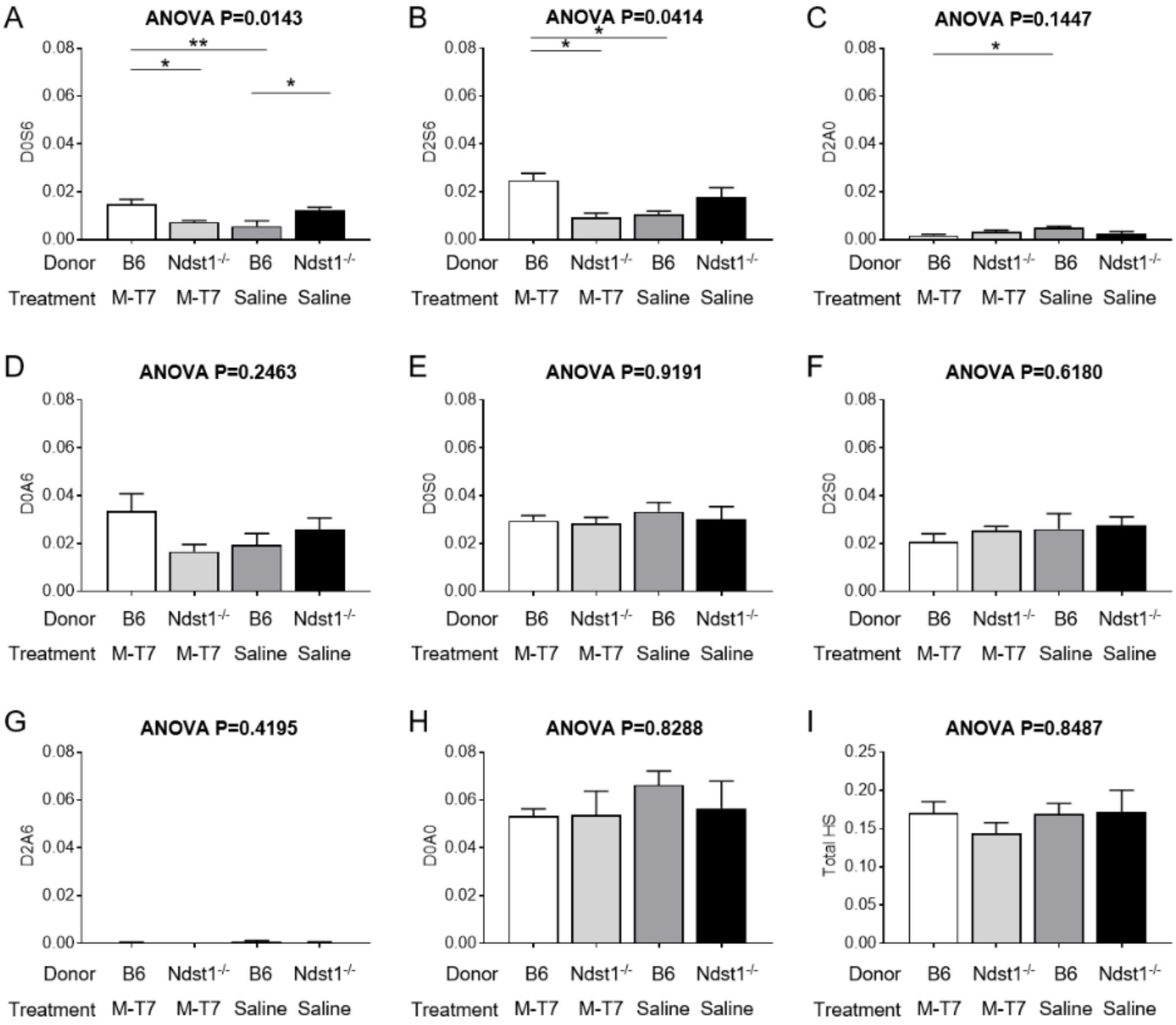

Supplemental Figure S4

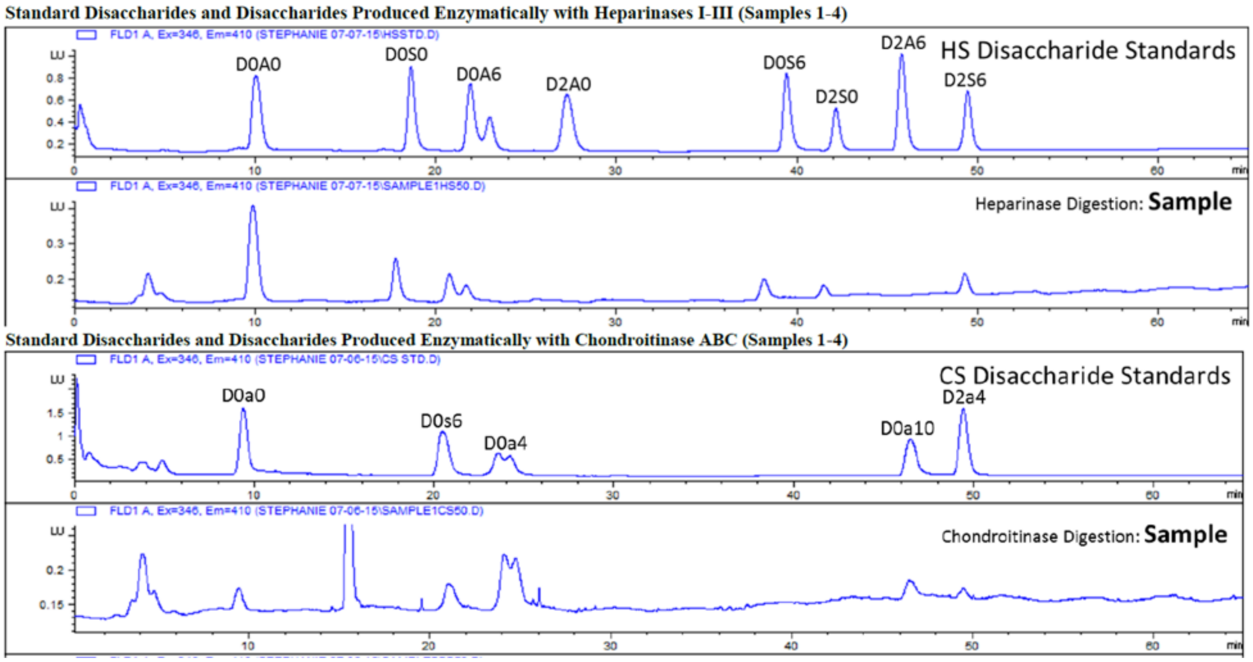

Supplementary Figure 3: Representative HPLC chromatograms for disaccharide analyses.

Supplementary Table 1 – Gene list in gene expression array.

SYMBOL	DESCRIPTION	GENE NAME
**ATF2**	Activating transcription factor 2	CRE-BP1/CREB2
**BAX**	BCL2-associated X protein	BCL2L4
**BCL2**	B-cell CLL/lymphoma 2	Bcl-2
**BCL2A1**	BCL2-related protein A1	ACC-1/ACC-2
**BCL2L1**	BCL2-like 1	BCL-XL/S
**NAIP**	NLR family, apoptosis inhibitory protein	BIRC1/NLRB1
**BIRC2**	Baculoviral IAP repeat-containing 2	API1/HIAP2
**BIRC3**	Baculoviral IAP repeat-containing 3	AIP1/API2
**BMP2**	Bone morphogenetic protein 2	BMP2A
**BMP4**	Bone morphogenetic protein 4	BMP2B/BMP2B1
**BRCA1**	Breast cancer 1, early onset	BRCAI/BRCC1
**CCL2**	Chemokine (C-C motif) ligand 2	GDCF-2/HC11
**CCL20**	Chemokine (C-C motif) ligand 20	CKb4/LARC
**CCND1**	Cyclin D1	BCL1/D11S287E
**CD5**	CD5 molecule	LEU1/T1
**CDK2**	Cyclin-dependent kinase 2	p33(CDK2)

**CDKN1A**	Cyclin-dependent kinase inhibitor 1A (p21, Cip1)	CAP20/CDKN1
**CDKN1B**	Cyclin-dependent kinase inhibitor 1B (p27, Kip1)	CDKN4/KIP1
**CDKN2A**	Cyclin-dependent kinase inhibitor 2A (melanoma, p16, inhibits CDK4)	ARF/CDK4I
**CDKN2B**	Cyclin-dependent kinase inhibitor 2B (p15, inhibits CDK4)	CDK4I/INK4B
**CEBPB**	CCAAT/enhancer binding protein (C/EBP), beta	C/EBP-beta
**CSF2**	Colony stimulating factor 2 (granulocyte-macrophage)	GMCSF
**CXCL9**	Chemokine (C-X-C motif) ligand 9	CMK/Humig
**CYP19A1**	Cytochrome P450, family 19, subfamily A, polypeptide 1	ARO/ARO1
**EGR1**	Early growth response 1	AT225/G0S30
**EN1**	Engrailed homeobox 1	Engrailed 1
**FAS**	Fas (TNF receptor superfamily, member 6)	ALPS1A/APO-1
**FASLG**	Fas ligand (TNF superfamily, member 6)	APT1LG1/CD178
**FASN**	Fatty acid synthase	FAS/OA-519
**FN1**	Fibronectin 1	CIG/DKFZp686F10164
**FOS**	V-fos FBJ murine osteosarcoma viral oncogene homolog	AP-1/C-FOS
**FOXA2**	Forkhead box A2	HNF3B/TCF3B
**GADD45A**	Growth arrest and DNA-damage-inducible, alpha	DDIT1/GADD45
**GREB1**	GREB1 protein	KIAA0575
**GYS1**	Glycogen synthase 1 (muscle)	GSY/GYS
**HK2**	Hexokinase 2	DKFZp686M1669/HKII
**HOXA1**	Homeobox A1	BSAS/HOX1
**HSF1**	Heat shock transcription factor 1	HSTF1
**HSPB1**	Heat shock 27kDa protein 1	CMT2F/DKFZp586P1322
**HSP90AA2**	Heat shock protein 90kDa alpha (cytosolic), class A member 2	HSP90ALPHA/HSPCA
**ICAM1**	Intercellular adhesion molecule 1	BB2/CD54
**IGFBP3**	Insulin-like growth factor binding protein 3	BP-53/IBP3
**IKBKB**	Inhibitor of kappa light polypeptide gene enhancer in B-cells, kinase-B	IKK-beta/IKK2
**IL1A**	Interleukin 1, alpha	IL-1A/IL1
**IL2**	Interleukin 2	IL-2/TCGF
**IL4**	Interleukin 4	BCGF-1/BCGF1
**IL4R**	Interleukin 4 receptor	CD124/IL4RA
**IL8**	Interleukin 8	CXCL8/GCP-1
**IRF1**	Interferon regulatory factor 1	IRF-1/MAR
**JUN**	Jun oncogene	AP-1/AP1
**KLK2**	Kallikrein-related peptidase 2	KLK2A2/hK2
**LEF1**	Lymphoid enhancer-binding factor 1	DKFZp586H0919/TCF1ALPHA
**LEP**	Leptin	OB/OBS
**LTA**	Lymphotoxin alpha (TNF superfamily, member 1)	LT/TNFB
**MDM2**	Mdm2 p53 binding protein homolog (mouse)	HDMX/hdm2
**MMP10**	Matrix metallopeptidase 10 (stromelysin 2)	SL-2/STMY2

**MMP7**	Matrix metallopeptidase 7 (matrilysin, uterine)	MMP-7/MPSL1
**MYC**	V-myc myelocytomatosis viral oncogene homolog (avian)	MRTL/bHLHe39
**NFKB1**	Nuclear factor of kappa light polypeptide gene enhancer in B-cells 1	DKFZp686C01211/EBP-1
**NOS2**	Nitric oxide synthase 2, inducible	HEP-NOS/INOS
**NRIP1**	Nuclear receptor interacting protein 1	RIP140
**ODC1**	Ornithine decarboxylase 1	ODC
**PECAM1**	Platelet/endothelial cell adhesion molecule	CD31/PECAM-1
**PPARG**	Peroxisome proliferator-activated receptor gamma	CIMT1/NR1C3
**PRKCA**	Protein kinase C, alpha	AAG6/PKC-alpha
**PRKCE**	Protein kinase C, epsilon	PKCE/nPKC-epsilon
**PTCH1**	Patched homolog 1 (Drosophila)	BCNS/HPE7
**PTGS2**	Prostaglandin-endoperoxide synthase 2 (prostaglandin G/H synthase and cyclooxygenase)	COX-2/COX2
**RBP1**	Retinol binding protein 1, cellular	CRABP-I/CRBP
**SELE**	Selectin E	CD62E/ELAM
**SELPLG**	Selectin P ligand	CD162/CLA
**TANK**	TRAF family member-associated NFKB activator	I-TRAF/TRAF2
**TCF7**	Transcription factor 7 (T-cell specific, HMG-box)	TCF-1
**TERT**	Telomerase reverse transcriptase	EST2/TCS1
**TFRC**	Transferrin receptor (p90, CD71)	CD71/TFR
**PMEPA1**	Prostate transmembrane protein, androgen induced 1	STAG1/TMEPAI
**TNF**	Tumor necrosis factor (TNF superfamily, member 2)	DIF/TNF-alpha
**TP53**	Tumor protein p53	LFS1/TRP53
**TP53I3**	Tumor protein p53 inducible protein 3	PIG3
**VCAM1**	Vascular cell adhesion molecule 1	CD106/DKFZp779G2333
**VEGFA**	Vascular endothelial growth factor A	MVCD1/VEGF
**WISP1**	WNT1 inducible signaling pathway protein 1	CCN4/WISP1c
**WNT1**	Wingless-type MMTV integration site family, member 1	INT1
**WNT2**	Wingless-type MMTV integration site family member 2	INT1L1/IRP
**B2M**	Beta-2-microglobulin	B2M
**HPRT1**	Hypoxanthine phosphoribosyltransferase 1	HGPRT/HPRT
**RPL13A**	Ribosomal protein L13a	RPL13A
**GAPDH**	Glyceraldehyde-3-phosphate dehydrogenase	G3PD/GAPD
**ACTB**	Actin, beta	PS1TP5BP1
**HGDC**	Human Genomic DNA Contamination	HIGX1A
**RTC**	Reverse Transcription Control	RTC
**RTC**	Reverse Transcription Control	RTC
**RTC**	Reverse Transcription Control	RTC
**PPC**	Positive PCR Control	PPC
**PPC**	Positive PCR Control	PPC
**PPC**	Positive PCR Control	PPC
